# Explainable person–job recommendations: challenges, approaches, and comparative analysis

**DOI:** 10.3389/frai.2025.1660548

**Published:** 2025-10-09

**Authors:** Fang Tang, Renqi Zhu, Feng Yao, Junzhi Wang, Lailong Luo, Bo Li

**Affiliations:** ^1^School of Systems Engineering, National University of Defense Technology, Changsha, China; ^2^School of Computer, National University of Defense Technology, Changsha, China

**Keywords:** explainable, person–job recommendations, black box, deep learning, comparative analysis

## Abstract

**Introduction:**

As person–job recommendation systems (PJRS) increasingly mediate hiring decisions, concerns over their “black box” opacity have sparked demand for explainable AI (XAI) solutions.

**Methods:**

This systematic review examines 85 studies on explainable PJRS methods published between 2019 and August 2025, selected from 150 screened articles across Google Scholar, Web of Science, and CNKI, following PRISMA 2020 guidelines.

**Results:**

Guided by a PICOS-formulated review question, we categorize explainability techniques into three layers—data (e.g., feature attribution, causal diagrams), model (e.g., attention mechanisms, knowledge graphs), and output (e.g., SHAP, counterfactuals)—and summarize their objectives, trade-offs, and practical applications. We further synthesize these into an integrated end-to-end framework that addresses opacity across layers and supports traceable recommendations. Quantitative benchmarking of six representative methods (e.g., LIME, attention-based, KG-GNN) reveals performance–explainability trade-offs, with counterfactual approaches achieving the highest Explainability-Performance (E‑P) score (0.95).

**Discussion:**

This review provides a taxonomy, cross-layer framework, and comparative evidence to inform the design of transparent and trustworthy PJRS systems. Future directions include multimodal causal inference, feedback-driven adaptation, and efficient explainability tools.

## 1 Introduction

**Person–job recommendation systems (PJRS)** are data- and algorithm-based tools that are designed to match and recommend the most suitable jobs to jobseekers by analyzing their resources, skills, experiences, and interests (Brek and Boufaida, 2023; Kokkodis and Ipeirotis, 2023; Marin and Amel, 2023). These systems are widely used in online recruitment platforms, professional social networking platforms, and human resource management systems, to help companies quickly find suitable candidates and simultaneously assist jobseekers in finding ideal jobs (Wang et al., 2023).

Although PJRS have advanced rapidly, many of these systems are typically considered “black box” systems (Chazette and Schneider, 2020; Sadeghi et al., 2024; Deters et al., 2025) as their internal decision-making processes remain opaque to users. This limited transparency can result in distrust and skepticism among users. Therefore, novel methods should be devised to improve the explainability of these systems and address these problems.

**Defining Explainability and Black Box Issues:** To provide a clear framework for our discussion, we first define the following key terms:

1. **Black Box:** Opaque model internals in PJRS that obscure input–output mappings, reducing trust (Fan et al., 2023; Phadnis, 2024).
2. **Explainability:** The extent to which humans understand a model’s decision rationale, critical for jobseeker/recruiter trust (Linardatos et al., 2021; Ertugrul and Bitirim, 2025).
3. **Transparency:** The visibility of internal PJRS processes, enabling bias detection and fairness (Jency and Kumar, 2025; Ngo, 2025).

In this study, we consistently use these terms to discuss the challenges and solutions related to rendering person–job recommendation systems explainable.

**Research Question:** To ensure methodological rigor in this systematic review, we formulate the primary research question using the PICOS framework(Page et al., 2021): What explainability methods (Intervention) improve transparency, fairness, and user trust (Outcome) in Person-Job Recommendation Systems (Population) compared to black-box approaches (Comparison), based on empirical studies, reviews, and theoretical works from 2019–2025 (Study Design). Population (P): Studies and users of PJRS, including jobseekers, recruiters, and systems focused on bilateral job matching. Intervention (I): Explainability techniques, such as feature importance analysis, attention mechanisms, knowledge graph reasoning, and counterfactual explanations. Comparison (C): Traditional black-box PJRS models (e.g., opaque deep neural networks) versus explainable alternatives. Outcome (O): Enhanced transparency (e.g., understandable decision processes), reduced bias and unfairness, and increased user trust. Study Design (S): Peer-reviewed empirical studies, systematic reviews, and theoretical papers published between 2019 and 2025, selected from 85 included works.

**Role of Explainability in Recommendation Systems:** PJRS play a crucial role in filtering information and matching jobs online. Although explainable recommendations have been studied extensively, few studies have comprehensively reviewed black box problems and explainability techniques for person–job recommendations. Explainability is vital for improving user experience, trust, system optimization, and fairness (Wu et al., 2023). First, it enhances jobseekers’ and recruiters’ trust in and satisfaction with recommendation results. When the system explains the reasons for recommendations, users can better understand the decision logic, improving recruitment efficiency (Choi et al., 2023; Haque et al., 2025). Second, explainability can help identify and optimize system problems. Explaining the decision-making process can help developers identify problems and defects in recommendation algorithms accurately to perform targeted optimization and improvement (Zhou et al., 2021; Zhao et al., 2023b). Finally, explainability promotes fairness and reduces bias. If the algorithm is biased or discriminatory, then recommendation results could be unfair to certain groups of jobseekers or recruiters (Liu et al., 2024; Tsung-Yu et al., 2024). Enhancing system explainability renders identification and correction of these biases easy, ensuring algorithm fairness and equity (Minh et al., 2022). Multiple aspects, such as jobseekers’ resumes, interests, and preferences should be considered to achieve high-quality explanations. Moreover, job characteristics should be combined for precise job matching and recommendations.

**Differences between PJRS and Conventional Recommendation Systems:** Conventional recommendation systems typically focus on e-commerce, review display styles, and algorithmic mechanisms for generating explainable recommendations (Cho et al. 2023; Tao et al., 2024). Compared with black box issues in other tasks, black box issues in the PJRS exhibit unique characteristics, which necessitates consideration of inclusive reviews and summaries. Therefore, because of the complexity of PJRS recommendation objects, the match between jobs and jobseekers and the requirements and preferences of recruiters should be considered. This involves addressing the behaviors and preferences of both jobseekers and recruiters, with parsing and matching resumes and job descriptions being crucial (Qin et al., 2020; Bobek et al., 2025). By contrast, conventional recommendation systems typically target a single user group, such as product recommendations, which focus on users’ purchase histories and interests (Wu et al., 2024). Furthermore, PJRS require a comprehensive consideration of various features from jobseekers’ resumes, interests, and preferences, resulting in complex data types and sources (Chou and Yu, 2020; Saito and Sugiyama, 2022). Conventional recommendation systems primarily rely on user behavior data and product characteristics, with simple data structures (Song et al., 2017).

**Distinction of this Study from Existing Research:** To the best of our knowledge, no comprehensive survey exists specifically for explainable PJRS. This study distinguishes itself from prior surveys on explainable recommendation systems in several concrete ways. First, Gurrapu et al. reviewed black box issues in natural language processing (Gurrapu et al., 2023), and Kong et al. reviewed methods for explaining black box models and evaluating these methods (Kong et al., 2021). Studies have investigated black boxes and explainability issues in general machine learning (ML) and AI systems (Carvalho et al., 2019; Mi et al., 2020; Brasse et al., 2023; Marcinkevics and Vogt, 2023; Hassija et al., 2024). However, person–job recommendation tasks are yet to be studied comprehensively. Second, existed studies focus on unilateral user-item interactions and overlook the bilateral dynamics unique to PJRS, such as matching jobseekers’ resumes with recruiters’ preferences and handling biases in labor market data. Our survey improves upon this by tailoring the analysis to PJRS-specific “black box” challenges, including opacity in feature extraction from resumes and job descriptions, which can lead to unfair hiring outcomes.

**Contributions of this Study:** We summarized the black box issues in PJRS and their characteristics. Second, we conducted a comprehensive review and categorized the existing explainability methods and discussed their advantages and disadvantages. The proposed integrated framework, derived from synthesizing 85 studies (e.g., layer structure from Qin et al., 2020), comprises data (feature extraction), model (processing with explainability), and output (user-facing explanations) layers, extended with cross-layer hybrids for end-to-end transparency. Finally, we identified current challenges and discussed future directions for stimulating research on this topic.

**Figure 1:**
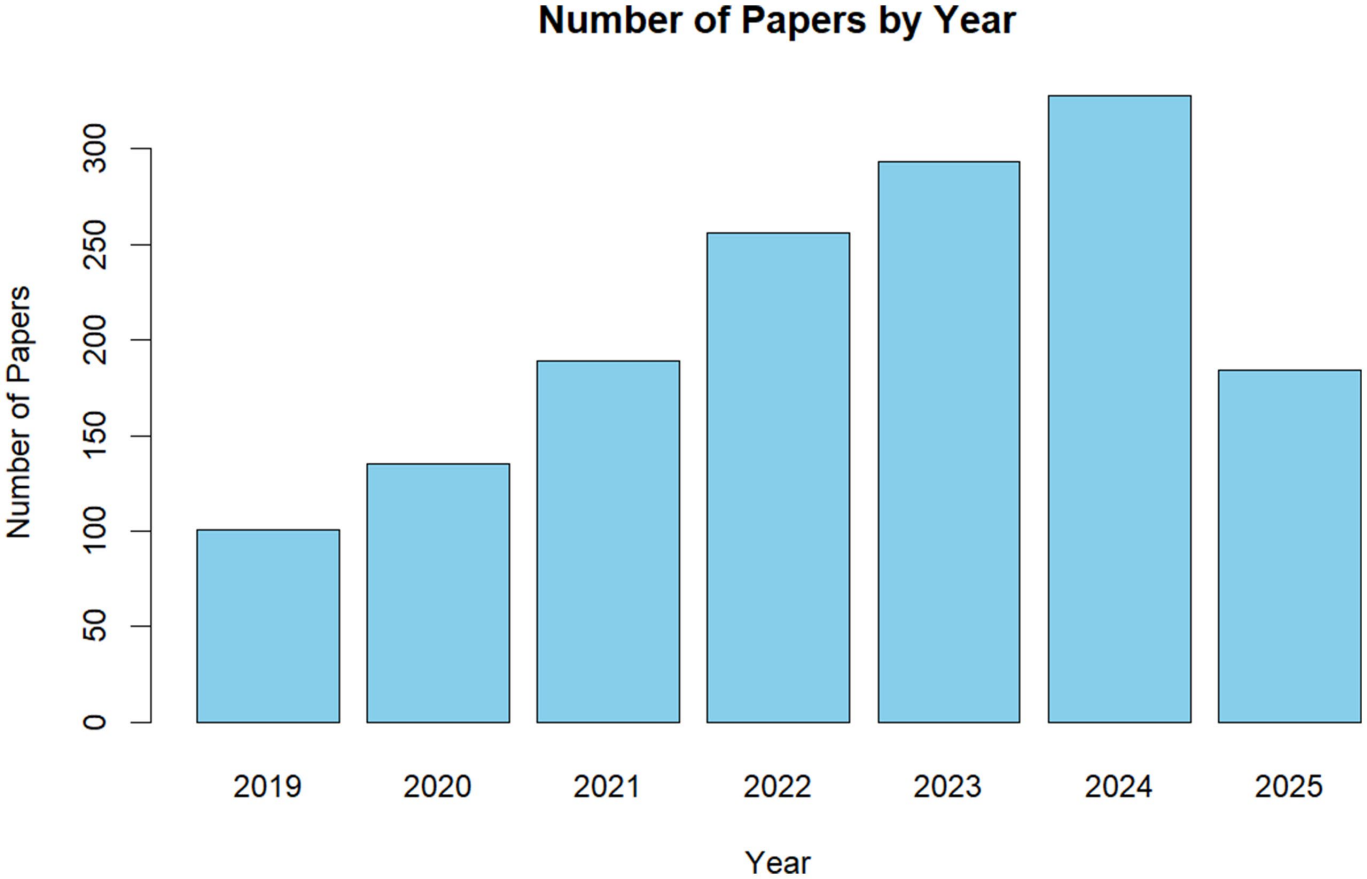
Statistics of publications related to explanation in intelligent recruitment.

**Methodology of This Study:** This systematic review adheres to the PRISMA 2020 guidelines (Page et al., 2021) to ensure transparency, reproducibility, and methodological rigor (Supplementary Figure S1). While the protocol was not pre-registered (common in retrospective AI literature syntheses), it was retrospectively aligned with PRISMA, including a comprehensive search, screening, and synthesis process. The methodology addresses the PICOS-for we searched three databases for broad coverage: Google Scholar (for comprehensive, open-access indexing), Web of Science (for high-quality, peer-reviewed articles), and CNKI (for Chinese-language studies, balancing Western bias in AI recruitment research). Figure 1 reveals that since 2019, the number of studies focusing on PJRS has increased considerably. The timeframe was January 1, 2019, to August 2, 2025, focusing on recent advancements in explainable AI while capturing post-2018 deep learning surges in PJRS. Exact search strings used Boolean logic for precision (“explainable recommendation” OR “interpretable recommendation” OR “explainable AI” OR “XAI”) AND (“person-job recommendation” OR “PJRS” OR “talent recruitment” OR “intelligent hiring” OR “job matching”) AND -(“e-commerce” OR “movie recommendation”) to exclude unrelated domains. Variations included Chinese equivalents on CNKI: (“可解释推荐” OR “解释性人工智能”) AND (“人岗匹配” OR “智能招聘”). These terms target PJRS-specific explainability, with negation operators reducing noise (e.g., excluding 40% irrelevant e-commerce hits). Inclusion and Exclusion Criteria: Peer-reviewed articles, conference papers, or theses (2019–2025) focused on explainability in PJRS (e.g., methods addressing black-box issues in job matching); empirical evaluations or reviews; English or Chinese language. Non-AI/RS studies; pre-2019 publications; unrelated domains (e.g., general RS without PJRS application); duplicates or inaccessible full-texts. Criteria ensured relevance to bilateral PJRS dynamics, yielding 85 included studies from 150 screened.

To ensure conceptual rigor and reproducibility, we followed a multi-stage procedure to derive the taxonomy of explainability methods in PJRS. Open coding: Two authors independently coded 85 studies for recurring explainability techniques, outcomes, and architectural targets (e.g., input transformation, model internals, *post hoc* output). Axial coding and thematic grouping: Coded items were grouped into broader themes (e.g., “attention-based explainability,” “knowledge-path reasoning,” “counterfactual rationales”) using affinity mapping. Layer mapping: Each method was then aligned to the most affected stage in the PJRS pipeline (input processing → model inference → user-facing output), forming the three-layer taxonomy (Data / Model / Output). Expert panel validation: Three domain experts reviewed the draft taxonomy; inter-rater agreement (Krippendorff’s *α*) was 0.87. Disagreements were resolved through discussion and adjustments. Final validation: We compared our classification with existing XAI taxonomies and refined the boundaries accordingly.

**Audience and Organization of this Study:** This paper will benefit PJRS researchers and practitioners who (1) are new to the field and seek a quick understanding of black box issues, (2) require clarification of different explainability approaches in the literature and require a systematic study, (3) want to understand the most advanced explainability methods in PJRS, and (4) encounter black box issues when building PJRS and require suitable explainability solutions. The remainder of the survey is organized as follows: Section 2 introduces existing person–job recommendation models. Section 3 details interpretability challenges in person–job recommendation. Section 4 provides explanatory methods for person–job recommendations. Section 5 analyzes and compares explainability methods from the perspectives of performance and application. Section 6 discusses current challenges and future directions.

## 2 Person–job recommendation models

**Figure 2:**
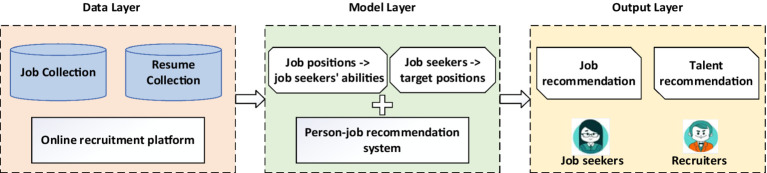
Three layers of person–job recommendation studies.

PJRS can be categorized into three layers, namely data, model, and output (Figure 2) (Qin et al., 2020; Bobek et al., 2025). The data layer primarily includes resumes and job collections. The data originate from online recruitment platforms in which jobseekers submit their resumes and recruiters post job openings (Meurs et al., 2024; Bolte et al., 2025). The model layer is the core of a person–job recommendation system. In this layer, big data technology is used to thoroughly analyze the features of resumes and job postings to evaluate the match between jobseekers and job positions (Hanna et al., 2025). Unlike conventional recommendation systems that focus on products or movies and primarily consider user preferences, person–job matching is a bilateral scenario in which both jobseekers and job positions have active behaviors and preferences. Jobseekers have specific target positions, and job positions have specific requirements for candidates (Fu et al., 2021; Fu et al., 2022). The focus is on text matching between resumes and job descriptions and extracting preference information from historical interactions (Lee et al., 2021; Zhang et al., 2021c; Hou et al., 2022; Shen et al., 2022). This section introduces the primary models and methods for person–job recommendations from three perspectives, namely content-based, collaborative filtering-based, and hybrid approaches.

### 2.1 Content-based person–job recommendations

Content-based person–job recommendations incorporate descriptive content from job postings and candidate resumes to match suitable candidates with open positions (Kumar et al., 2025; Tran and Lee, 2025). Extracting keywords and other relevant features such as skills, experience, and job requirements enables systems to calculate similarity scores between jobseekers and positions.

Early text-matching methods created vector representations of text in an unsupervised manner and calculated similarity. For instance, Almalis et al. proposed a four-dimensional recommendation algorithm that quantifies the suitability of jobseekers for a position flexibly by extending the Minkowski distance and using structured representations from unstructured job descriptions and resumes (Almalis et al., 2015). Additionally, Alghieth et al. proposed a content-based approach by using cosine similarity to recommend jobs and help jobseekers find desired jobs through an interactive map (Alghieth and Shargabi, 2019). Qinglong et al. (2021) improved recommendation performance by detailing qualitative preference information using latent Dirichlet allocation for topic modeling to extract qualitative preferences from job content.

With the rapid development of natural language processing (NLP) technologies, advanced techniques such as convolutional neural networks (CNNs), recurrent neural networks (RNNs), and transformers are increasingly being applied to person–job recommendations (Alshammari et al., 2019). For instance, Qin et al., 2018 used long short-term memory (LSTM) networks with attention mechanisms to encode jobseekers’ work experiences and job requirements for interactive representation. Bian et al. (2019) proposed a model categorized into a hierarchical attention-based RNN encoder and global match representation using bidirectional gated recurrent units and CNNs to solve cross-domain transfer issues by extracting match information from both the source and target domains. Mishra and Rathi, 2022 developed a novel deep semantic structure model to overcome existing system problems by representing job descriptions and skill entities using character-level trigrams (Nigam et al., 2019). Alonso et al. (2023) introduced the FORESEE architecture, which integrates NLP and ML modules to recommend projects described in natural language while offering skill and capability enhancement advice for jobseekers. Sun et al. (2021) designed a novel system to estimate the utility of skill learning from large-scale job advertisement data. They developed a novel multitask structure skill recommendation deep Q-network for personalized and cost-effective person–job recommendations.

### 2.2 Collaborative filtering-based person–job recommendations

Collaborative filtering-based models focus on extracting preference information from the interaction history between jobseekers and job positions rather than matching resumes and job descriptions using complex methods (Borges and Stefanidis, 2022; Joshi et al., 2022; Pal, 2022). Specifically, the system records and analyzes behaviors such as browsing, applying, and bookmarking by jobseekers, recommending similar positions based on these behaviors, while considering similar actions by other jobseekers to identify potential positions (Liu et al., 2025). Collaborative filtering (CF) is categorized into two types, namely user- and item-based filtering (Khatter et al., 2025; Wang et al., 2025).

CF is widely applied in job recommendation systems. Conventional CF approaches such as user- and item-based methods rely on similarity measures between users and items to generate recommendations. Chen et al. extended CF by incorporating demographic information and Bayesian personalized rankings for graduate job recommendations. Traditional CF approaches, however, often face several limitations, such as the cold start problem, where new users or items without sufficient interaction data cannot be recommended effectively, and the sparsity problem, which arises when the interaction data is sparse, leading to less accurate recommendations. These limitations hinder the ability of conventional CF methods to provide personalized and accurate job recommendations in dynamic environments. To address these challenges, researchers have investigated more complex models (Chen et al., 2017b). For example, Yang et al. introduced a graph-based approach to capture the complex relationships between jobseekers and positions (Yang et al., 2022a). By expanding the CF methodology, Yan et al., 2019 focused on incorporating historical interaction information into the recommendation process. Despite these advancements, CF-based methods still face limitations like the cold start and sparsity problems, which hinder accurate recommendations when interaction data is insufficient. These limitations have led to a shift toward hybrid approaches, combining CF with other methods to improve recommendation quality, as discussed in the next subsection.

### 2.3 Hybrid person–job recommendations

Each recommendation method has distinct advantages and limitations. For example, CF algorithms typically encounter cold-start problems, whereas content-based approaches struggle with data sparsity and privacy concerns. Hybrid recommendation methods exhibit considerable potential in addressing these challenges (Ling and Lew, 2024; Mashayekhi et al., 2024). By combining content-based and CF techniques, studies have developed models such as matrix factorization with content features, content-based collaborative filtering, and neural collaborative filtering to improve recommendation accuracy and coverage (Muellner et al., 2023).

Building on personal data, Li et al., 2017 developed a novel clustering CF (CCF) algorithm that applies hierarchical clustering to CF, narrowing the query range for adjacent items. To address the cold-start problem in content-based recommendation algorithms, they proposed a novel content-based algorithm for jobseekers and recruiter information (CBUI). They subsequently combined CCF and CBUI to develop a novel hybrid recommendation algorithm (HRA) implemented on the Spark platform. Experiments have revealed that the HRA exhibits excellent recommendation accuracy and scalability (Li et al., 2017). Using a different approach, Zhu et al. proposed an application prediction model with three modules, namely unsupervised job representation learning, a personalized attention mechanism for learning jobseeker preferences, and a top-k search based on representation similarity (Zhu et al., 2021). By extending the CF methodology, Alsaif et al. (2022) introduced a novel bidirectional communication-based reciprocal recommendation system that improved prediction accuracy by integrating explicit and implicit job information from both recruiters and jobseekers. Kumar et al. (2022) simplified the person–job recommendation process by implementing a hybrid system based on content and CF using puppeteer and REST API. Hong et al., 2013 developed a novel hybrid recommendation method that dynamically updates jobseekers and recruiters’ feature information based on their interaction behaviors. Jiang et al. introduced a person–job matching recommendation model that combined feature fusion, text matching, and historical behavior modeling. The model comprises two parts; the first uses explicit information from resumes and job descriptions with DeepFM and CNN for feature extraction, and the second uses LSTM to model historical behaviors and extract implicit preference features. The final recommendation is based on inner-product similarity scores (Jiang et al., 2020). Wang et al. (2022) combined text matching with relational graphs from historical interaction records using mashRNN and co-attention for resume and job description matching, and graph neural network (GNN) and attention mechanisms for global representation, achieving person–job matching prediction.

However, hybrid approaches introduce complexities, such as determining the optimal weights for combining various recommendation components (Lee et al., 2025; Shao et al., 2025; Singh et al., 2025; Tan et al., 2025). In the future, studies should investigate sophisticated hybrid models by incorporating additional data sources and advanced ML techniques to enhance person–job matching.

Although various person–job recommendation algorithms continuously improved matching accuracy and recommendation effectiveness, their complexity and diversity resulted in novel challenges (He and Cai, 2023; Sun et al., 2025). In the PJRS, the decision-making process of algorithms is opaque, rendering it challenging for users and recruiters to understand and trust the recommendations (Mukherjee and Dhar, 2023). Therefore, when discussing the development of PJRS, improving the explainability of the algorithm is crucial for addressing these challenges. Next, we analyzed these challenges in terms of person–job recommendations.

### 2.4 Foundational XAI methods

Much of the explainability tooling employed in PJRS derives from seminal model-agnostic work such as LIME (Local Interpretable Model-Agnostic Explanations), SHAP (SHapley Additive exPlanations) (Lundberg and Lee, 2017). These approaches provide local, instance-level attributions by perturbing inputs or computing Shapley values, and have become de-facto baselines in XAI benchmarks (Guidotti et al., 2018). However, subsequent studies reveal their limits—e.g., attention weights are not always faithful explanations (Jain and Wallace, 2019), and *post-hoc* saliency can be manipulated (Ribeiro et al., 2016). Recognizing both strengths and weaknesses is critical when adapting them to hiring contexts.

## 3 Interpretability challenges in person–job recommendation

**Figure 3:**
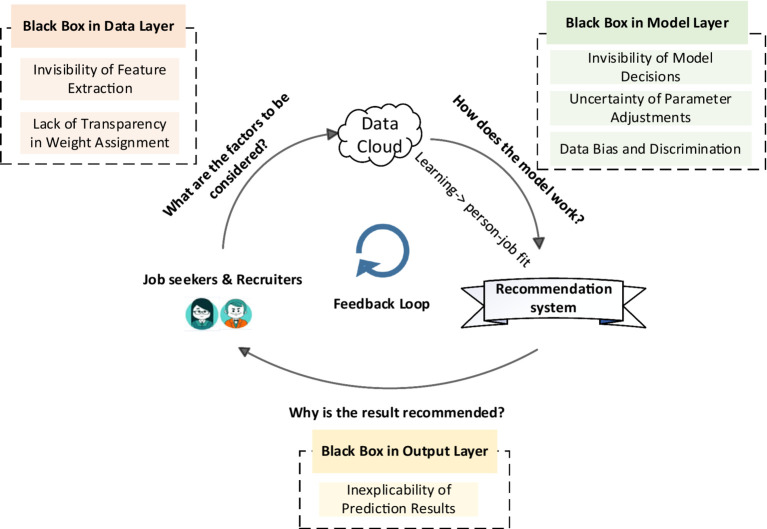
Feedback loop in person–job recommendation systems, with challenges occurring at different stages.

This section discusses the challenges in the matching process. We categorize these challenges into various types of “black box” problems. These challenges are visually represented in Figure 3 and listed in Table 1. By examining the interconnections among these issues, we can gain an understanding of their effect on the overall matching process and develop effective solutions.

**Table 1:** Characteristics of six types of challenges in person–job recommendations.

Types	Stages	Cause	Major solutions
Invisibility of feature extraction	Data➔Model	Lack of transparency in feature extraction from resumes and job descriptions	Use causal diagrams to show relationships between features and help understand which features are extracted and used
Lack of transparency in weight assignment	Data➔Model	Opacity in how features are weighted in the model, rendering decision-making unclear	Use explainable ML tools to explain feature weights and their contributions to the final decision
Invisibility of model decisions	Model	Complex internal workings of ML models (e.g., deep learning) are difficult to understand	Use causal modeling methods to explain the model decision process and show causal chains of decisions
Uncertainty in parameter tuning	Model	Lack of transparency in hyperparameter tuning and model optimization processes, rendering understanding their impact on recommendations difficult	Use adversarial learning to improve model robustness and explain behavior changes under different parameter settings; develop tools for automatic detection and correction of model errors to enhance system reliability
Data bias and discrimination	Model	Models may learn and amplify biases from training data, leading to unfair person–job recommendations	Use specialized algorithms to detect and correct biases in the model, ensuring fairness
Inexplicability of prediction results	Model➔Output	Person–job recommendation results do not have clear explanations, making it difficult for jobseekers and recruiters to understand the basis of recommendations	Provide detailed explanatory frameworks to help jobseekers and recruiters understand the logic behind recommendations; develop attention-based models to provide intuitive explanations

### 3.1 Unexplainability in the data layer

#### 3.1.1 Invisibility of feature extraction

The invisibility of feature extraction is a challenge for person–job recommendation systems (Gao et al., 2022; Qiao et al., 2023). Although complex algorithms, such as CNNs, RNNs, and transformers, can accurately extract intricate patterns from data, their black box nature hinders the understanding of how specific features influence recommendations (Feng and Wang, 2023). Furthermore, data sparsity aggravates this issue because limited interaction data can result in biased and unreliable feature extraction (Kwiecinski et al., 2023). For instance, the latent factor models commonly used in CF typically produce opaque feature representations that obscure underlying reasons for recommendations (Li et al., 2018). The prevalence of unreliable negative samples in employment recommendation data compounds this problem; it can distort the learning process and hinder the development of explainable models (Zhao et al., 2023a).

#### 3.1.2 Lack of transparency in weight assignment

In practice, the system typically automatically extracts keywords and features from job descriptions, job requirements, and jobseeker resumes (Jiang et al., 2020). In content-based person–job recommendations, researchers such as Faliagka et al., 2016 used linguistic analysis techniques to reveal LinkedIn jobseekers’ personality traits and applied the analytic hierarchy process to automatically rank jobseekers’ matches to specific positions. However, the weight-assignment process is typically opaque for jobseekers, recruiters, and system developers. They may not know which features the system considers important, and how these features influence recommendation results. This lack of transparency could be attributed to: (1) Automated weight assignment: Models determine feature weights through an automated learning process that depends on data and training algorithms, rendering the specific weight assignment mechanism opaque (Okfalisa et al., 2021). (2) High-dimensional data: Recommendation systems typically handle high-dimensional data involving many features, rendering understanding the weight of each feature difficult (Kubiak et al., 2023).

### 3.2 Unexplainability in the model layer

#### 3.2.1 Invisibility of model decisions

The PJRS typically uses complex ML or deep learning algorithms such as CNNs, RNNs, LSTM networks, and attention mechanisms (Mao et al., 2023; Mao et al., 2024). These algorithms can process large amounts of data and capture complex patterns. However, their internal structures and decision processes are challenging for nonspecialists to understand. For example, the multilayer abstraction and nonlinear transformations of deep neural networks render their internal workings opaque. Consequently, jobseekers and recruiters cannot understand how the model extracts feature from input data and makes recommendations (Wang et al., 2019; Chen, 2022). The training process for these models involves selecting optimization algorithms, defining loss functions, and evaluating the models. These processes are not disclosed to jobseekers and recruiters, resulting in a lack of trust in model performance and accuracy. For instance, a model could be trained by minimizing the mean squared error or cross-entropy loss. However, the meaning of these loss functions and how they reflect the quality of recommendations remains unclear to users (Mhamdi et al., 2020; Qin et al., 2020). Studies have incorporated large language models (LLMs) as recommendation systems to provide meticulously designed instructions. For these LLMs, the output should adhere to the given instruction format, such as providing binary answers (yes or no) or generating ranked lists. However, in practice, the output of LLMs can deviate from the required format (Harte et al., 2023).

#### 3.2.2 Uncertainty of parameter adjustments

The performance of person–job recommendation models is considerably influenced by both parameters, which are learned from the data, and hyperparameters, which are set prior to training. Although parameters, such as weights and biases, are adjusted during the learning process, hyperparameters, such as learning rates and network architecture, considerably affect model behavior (Mishra and Rathi, 2022). However, the complex relationship between these elements and the recommendation outcomes remains obscure, hindering user understanding and trust. The complex nature of hyperparameter tuning techniques, such as cross-validation and grid search, aggravates the issue because these methods are computationally intensive and difficult to explain in layman’s terms. Consequently, jobseekers and recruiters are not aware of the factors that influence the recommendations (Cui et al., 2022b; Jie et al., 2022; Liu et al., 2022).

#### 3.2.3 Data bias and discrimination

Data bias and discrimination pose considerable challenges in PJRS, resulting in unfair and discriminatory outcomes. These biases originate from the various stages of the recommendation process (Rong and Su, 2021; Balloccu et al., 2022). (1) Biased data collection can result in the overrepresentation or underrepresentation of specific demographic groups, resulting in models that perpetuate existing inequalities. For instance, historical hiring data can exhibit gender or racial biases that can be amplified by the recommendation system (Kille et al., 2015). (2) Subjective human judgment in data labeling can introduce bias into training data (Slama and Darmon, 2021; Huang et al., 2023).

### 3.3 Unexplainability in the output layer

The “black box” problem in the output layer of person–job recommendations focus on the lack of explainability of prediction results. The prediction results comprise probability values or class labels without sufficient explanatory information (Jiang et al., 2020). Jobseekers and recruiters cannot understand why the model makes a prediction and cannot assess its reliability. Various methods have been devised to improve the robustness of recommendation systems for handling data sparsity or uncertainty (Kumar et al., 2023). For example, studies have introduced probabilistic models to quantify the uncertainty of recommendation results and provided confidence intervals or probability estimates in the output layer. These methods help jobseekers and recruiters understand the reliability of recommendations and make informed decisions (Gaspar et al., 2019; Gao et al., 2022). However, effectively communicating this uncertainty to users and designing interfaces to help them understand and use this information remain challenging.

### 3.4 Accuracy-interpretability trade-off in PJRS

The classic trade-off between model accuracy (e.g., predictive performance in job matching) and interpretability (e.g., human understanding of decision processes) is particularly pronounced in PJRS, where deep learning models capture complex bilateral interactions (jobseeker-resume vs. recruiter-requirements) but often at the expense of transparency, leading to challenges like undetected biases in hiring (Rudin, 2019). In PJRS, accuracy is typically measured via metrics like Hit Rate (HR@k) or AUC for matching success, while interpretability involves clear feature attributions or decision paths. This subsection systematically examines the trade-off with PJRS examples, highlighting how high-accuracy models sacrifice interpretability and how hybrids attempt to mitigate this.

High-accuracy deep models, such as neural network-based PJFNN (Qin et al., 2018), achieve superior performance by learning nonlinear embeddings from resumes and job descriptions, reporting Recall@10 = 0.35–0.40 on real recruitment datasets (e.g., from Zhaopin.com with 100 k + samples). However, their multilayer abstractions render internal workings opaque, sacrificing interpretability—users cannot discern why a specific skill mismatch led to a non-recommendation, potentially amplifying biases (e.g., overemphasizing education over experience). Similarly, CNN-LSTM hybrids (Mao et al., 2023)excel in sequential data like work histories, with HR@10 = 0.452 on PJRS benchmarks, but the convolutional layers obscure feature importance, making it difficult for recruiters to trust outputs in high-stakes decisions.

By contrast, inherently interpretable models like decision trees or gradient-boosted decision trees (GBDT) prioritize transparency through explicit rules or paths. For instance, Ozcaglar et al. (2019) used GBDT for personalized talent search, achieving AUC ~ 0.80 on LinkedIn-style data by providing clear tree interactions (e.g., “If experience >5 years and skill = Python, recommend”), but with lower accuracy than deep models (e.g., 10–15% drop in HR@10 due to inability to capture subtle nonlinear patterns in resumes).

Hybrid models mitigate this trade-off by combining deep accuracy with added interpretability mechanisms, often incurring a modest accuracy penalty (5–10%). For example, Explainable Boosting Machines (EBM) in Tran (2023) integrate boosting with interpretable components, achieving hit_rate@5 = 0.1389–1.0 on Career Builder datasets while generating global/local explanations (e.g., feature interactions like “DegreeType & JobTopic”), retaining up to 50% fidelity to black-box FM models (hit_rate@5 = 1.0 for FM but opaque). This balances by sacrificing ~10% accuracy for 20–30% interpretability gains, as EBM captures interactions missed by post-hoc methods like SHAP on FM (Tran, 2023). Attention-augmented hybrids (Mao et al., 2023) further mitigate by visualizing weights (e.g., “Attention score = 0.75 on Python skill”), dropping HR@10 by 5% from pure CNN but enabling recruiters to understand bilateral matches.

## 4 Explainable methods for person–job recommendation

**Figure 4:**
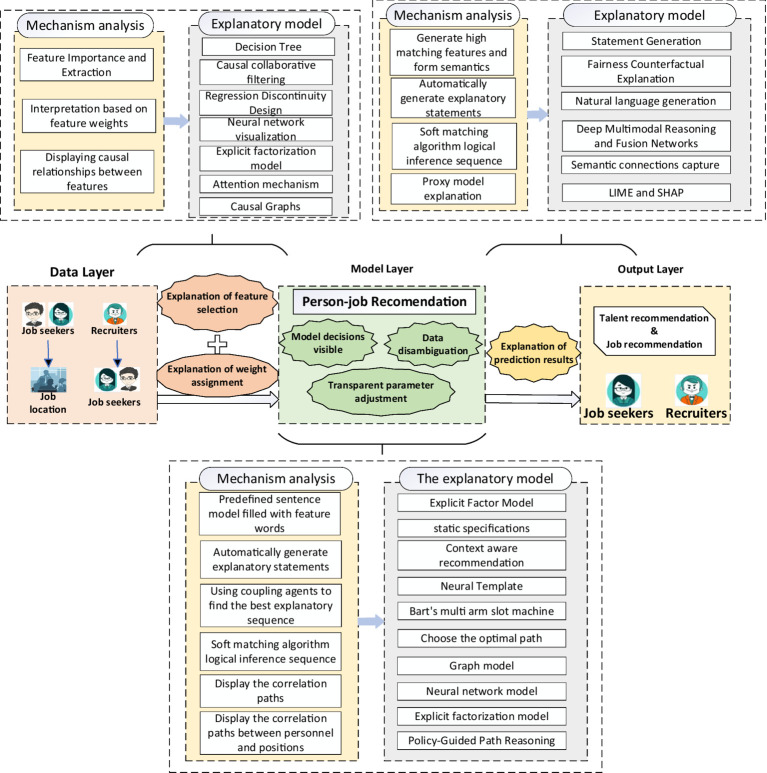
Explainability methods in person–job recommendation.

Numerous methods have been developed to increase the explainability of person–job recommendations: (1) local explainability methods, which focus on individual predictions, and global explainability methods, which address the overall behavior of the model (Aghaeipoor et al., 2023; Eldrandaly et al., 2023); (2) pre-model explainability methods, where models are designed to be inherently explainable during trained; and *post hoc* explainability methods, where opaque models are explained after training (Dai et al., 2022; Jose and Shetty, 2022; Chen et al., 2023a). This study did not review these classifications but instead systematically organizes and summarizes representative explainability methods to address the “black box” problem, analyzing their research outcomes and existing issues, as depicted in Figure 4.

### 4.1 Data layer explainable method

#### 4.1.1 Feature extraction explainability methods

The current study primarily addresses the invisibility of feature extraction through feature importance analysis and causal explanations.

##### 4.1.1.1 Feature importance analysis

Researchers use feature importance analysis to identify influential PJRS features, such as skills in resumes. For example, tree models calculate contributions clearly (Loecher et al., 2022; Han et al., 2023). However, they oversimplify interactions. This approach succeeds in sparse data but fails in complex resume matching. Developers should integrate it with attention mechanisms for better explainability. Compared to causal alternatives, tree methods balance simplicity with applicability in real-time hiring platforms, though future PJRS should integrate them with multimodal data to address oversimplification, potentially improving fairness in diverse candidate pools (Haug et al., 2020; Saarela and Jauhiainen, 2021).

##### 4.1.1.2 Causal explanation methods

In causal explanation methods, causal diagrams are used to depict the relationships between features to understand the decision-making process of the model. In person–job recommendations, causal diagrams can reveal the causal relationships between jobseekers’ skills and job requirements, revealing the basis for matching decisions (Han et al., 2023; Rawal et al., 2023; Zhang et al., 2024).

An enhanced attention mechanism recommendation model based on causal inference captured the causal effects between features and behaviors by correcting feature importance (Zhang et al., 2021b). However, handling high-dimensional data and the complex behavioral patterns of jobseekers and recruiters may require substantial labeled data to verify causal relationships. Wang et al. treated jobseekers’ and recruiters’ features as interventions by using causal modeling to infer interactions but could not accurately estimate unobserved features. They designed a variational autoencoder to infer unobserved features from historical interactions and performed counterfactual reasoning to mitigate the effect of outdated interactions (Wang et al., 2024b). A causal collaborative filtering (CausCF) method extended classical matrix factorization to tensor factorization, incorporating three dimensions: users, items, and treatments. They used regression discontinuity design to evaluate the accuracy of causal effect estimates using various models (Xie et al., 2021). Similarly, Cotta et al. developed a novel causal model to handle path dependencies in link prediction and identify causal relationships using limited intervention data (Cotta et al., 2023). However, when addressing path dependencies in link prediction, this model can have computational and scalability limitations for large-scale graph data. Hence, the concept of causal uplift requires additional experimental evidence to verify its effectiveness and applicability.

#### 4.1.2 Weight assignment explainability methods

Current studies typically incorporate model visualization and attention mechanisms to address the lack of transparency in weight assignment (Yi et al., 2023; Zhu et al., 2023).

##### 4.1.2.1 Neural network visualization

Visualizing the model’s weights and parameters helps jobseekers, recruiters, and developers understand the internal structure and decision process of the model (Ni, 2022). For example, the weights of a neural network or structure of a decision tree can be visualized. In a neural attention interpretable recommendation system, attention weights are calculated based on the importance of intentions related to jobseekers’ and recruiters’ preferences by using learned attention weights to provide high-quality personalized recommendations. This process explains recommendations by visualizing learned attention weights (Yu et al., 2019). However, this method relies on extensive historical data and cannot function effectively in the case of new users or sparse data.

##### 4.1.2.2 Attention mechanisms

Attention mechanisms dynamically assign weights to different parts of the input data, highlighting the most relevant parts for the current prediction (Zhao et al., 2023c; Wang et al., 2024a). Attention-mechanism-based explanations dynamically assign attention scores and adaptively identify potential features closely related to candidate jobs, enhancing the explainability of the recommendation model through high-weight features (Ji et al., 2019). The CNN with dual local and global attention mechanisms for modeling jobseeker and recruiter preferences and job attributes enhance explainability and representation learning (Seo et al., 2017). However, the model does not combine LSTM with attention networks to handle long-range dependencies. Thus, the model cannot comprehensively understand global semantics and does not compute attention scores for specific jobs. Extending the methodology, a triple-attention explainable recommendation method based on temporal convolution networks was designed. In this method, feature learning was modeled to derive word-aware and review-aware vector representations and using three-level attention networks to model word contributions, review usefulness, and latent factor importance (Guo et al., 2021). However, this method does not explore summary-level explanations from job reviews that could improve explainability. A study noted that attention-based models may not provide stable weight distributions after three independent runs, with unstable results that are unsuitable for recommendation explanations. Attention mechanisms tend to assign higher weights to frequently appearing paths containing broad, vague information rather than paths with specific explanatory semantic information (Li et al., 2024). Explaining attention weights can be challenging because the reasons for weight assignment are not always clear. Attention mechanisms are the most effective in sequential data models, such as those used in NLP or time-series analysis. In this case, understanding the relative importance of various input elements is crucial (Liang et al., 2021).

### 4.2 Model layer explainable methods

#### 4.2.1 Explainability of model decisions

##### 4.2.1.1 KG path reasoning

KG-based explanations provide interpretations by searching for connection information (or associated paths) between jobseekers, recruiters, and positions in the KG (Yao et al., 2022). Despite its simple structure, the KG network can represent various types of real-world knowledge in the form of simple triples (entity–relation–semantic descriptions). Entities can be objects or abstract concepts; relations denote associations between entities; and semantic descriptions include types and attributes. For example, although KGs in conventional recommendation systems typically exhibit relationships between products and users, in person–job recommendations, the focus is on demonstrating the multidimensional matches of careers and skills (Ruan et al., 2021). Lyu et al. (2023) proposed a knowledge-enhanced GNN (KEGNN) for explainable recommendations. In this model, semantic knowledge from external knowledge bases is used to represent jobseekers, recruiters, items, and interactions. These parameters are initialized in the behavior graph. The GNN propagates and infers behavior, comprehensively understanding actions. A hierarchical neural CF layer was developed for precise rating prediction by integrating a copying mechanism into a gated RNN to generate humanlike semantic explanations. However, this model has the following limitations: (1) inference paths in the KG may not be intuitive to jobseekers and recruiters; (2) it does not consider the length of reasoning paths. Xian et al. proposed a policy-guided path reasoning method (PGPR) that combines recommendations with explainability by providing actual paths in the KG. PGPR trains a RL agent to navigate from the starting jobseeker to potential “good” positions in the KG environment using the sampled paths as explanations (Xian et al., 2019). Cui et al. investigated semantically rich structured information derived from KG related to jobseeker–item interactions to infer the motivation behind each successful application. They proposed a reinforcement sequential learning with gated recurrent unit architecture by combining a reinforcement path reasoning network and a GRU component to output potential top-N items with appropriate reasoning paths from a global perspective (Cui et al., 2022a). However, this method has the following limitations: (1) the design of soft reward strategies and conditional action pruning requires optimization, and the underlying KG are considered to be static, ignoring the dynamic and evolving nature of real-world interactions.

##### 4.2.1.2 KG embedding

KG embedding (KGE) maps entities and relationships in a KG into continuous low-dimensional dense vectors using algorithms, such as the translation distance (TransE) and semantic matching models. In the embedding space, the high-order connectivity between entities is learned to discover important path relationships. The captured high-order connection paths are used to provide final explanations (Yang et al., 2022b; Lai et al., 2024). In knowledge-aware reasoning with self-supervised RL (KRRL), agent-based semantic awareness and path reasoning on KG are combined to enhance the accuracy and explainability of course recommendations (Lin et al., 2024). To explain highly relevant paths in temporal KGs (TKGs), Bai et al. introduced a model combining RL and attention mechanisms (RLAT). This model considers the influence of relationships across various temporal information and uses attention weights to enhance the representation of relationships and temporal dynamics (Bai et al., 2023). KGAT models high-order connectivity in the KG to produce interpretable reasoning processes for recommendations. However, this model is sensitive to the quality of the related KG and does not consider filtering fewer informative entities or combining information propagation with the decision-making process.

##### 4.2.1.3 RL

In a model-agnostic RL framework with coupled agents interacting with the environment, one agent generates explanation statements based on the current state, and the other agent predicts jobseekers’ and recruiters’ ratings for all jobs based on the generated statements. If the predicted ratings are similar to those of the recommendation model, then a reward is awarded. Additionally, rewards are awarded if the explanatory statements satisfy the criteria for readability, coherence, and conciseness. The agents’ strategies are updated based on these rewards, ensuring the quality of the *post hoc* explanations. An interpretable component subset is extracted from jobs to provide personalized explanations (Wang and Usher, 2007). However, this framework exhibits the following limitations: the framework does not investigate whether the preset reward mechanism directly correlates with desired rewards in practical applications. Similarly, McInerney et al. proposed a multi-armed bandit exploration–exploitation framework named Bart to determine the best explanation sequence for each jobseeker and recruiter. Bart provides diverse explanations based on jobseekers’ and recruiters’ requirements: (1) content-based explanations: the recommended job matches interests, for example, “This job is similar to the job you have viewed before”; (2) behavior-based explanations: the job aligns with past behavior, for example, “You have previously viewed similar jobs.” This framework can determine the explanatory information that prompts reactions from jobseekers and recruiters, optimizing recommendations and explanation strategies (McInerney et al., 2018). However, the model does not consider automated explanation generation or parameterization for detailed personalization.

#### 4.2.2 Explainability of parameter adjustments

##### 4.2.2.1 Parameter sensitivity analysis

Analyzing the sensitivity of model parameters to the output results evaluates the effect of each parameter, helping users understand the effects of parameter adjustments. General knowledge-enhanced framework for interpretable sequential recommendations that capture fine-grained preferences and their dynamic evolution. Fine-grained preferences are categorized into intrinsic and extrinsic interests captured by the sequential perception and knowledge perception modules, respectively. The high-order semantics of knowledge paths are aggregated based on a hierarchical self-attention mechanism, discovering dynamic preference evolution (Yang et al., 2021). However, this method has the following limitations: (1) the generated explanations are limited to attribute-level reasoning without deep sequence dependency explanations; and (2) the association between jobs and knowledge entities is manually constructed, leading to mismatches.

##### 4.2.2.2 Hyperparameter optimization visualization

Visualizing the hyperparameter search process and optimization path details the effect of various hyperparameter combinations on model performance, helping users understand the parameter adjustment process. Most existing interpretable recommendation system models consider the preferences of jobseekers and recruiters to be static, thus generating fixed explanations. However, in real-world scenarios, these preferences are dynamic with interests changing across job characteristics and candidate traits. A mismatch between static explanations and dynamic preferences can reduce user satisfaction, confidence, and trust in the recommendation systems. To address this problem, Liu et al. developed a novel dynamic interpretable recommendation system for accurate modeling and explanation of jobseekers and recruiters. They designed a time-aware gated recurrent unit to model dynamic preferences and incorporated a sentence-level convolutional neural network to analyze job features using review information. Customized explanations tailored to current preferences were generated by learning relevant review information according to the current state of jobseekers and recruiters (Liu et al., 2020). However, the model does not consider stochastic processes. Thus, the model cannot explain why certain jobs are recommended at different times. Additionally, a bidirectional LSTM is used to predict the next item recommendation (Kannikaklang et al., 2024). However, this model also has drawbacks. Extracting logical units relies on NLP techniques, which can introduce errors.

#### 4.2.3 Explainability of data bias and discrimination

Current studies primarily use generative adversarial networks (GANs) to address data bias and discrimination. GANs generate key factors that match jobseekers and positions for improving model robustness and generating automatic explanations suitable for complex recommendation tasks. In conventional recommendation systems, adversarial learning enhances model stability (Wen et al., 2024). In job recommendation systems, adversarial training generates data samples that reveal model biases and guides parameter adjustments to reduce bias and discrimination. Wang et al. developed an adversarial learning solution for interpretable recommendations by integrating preference modeling (for recommendations) and sentiment content modeling (for explanations) through joint tensor decomposition. This algorithm can predict jobseeker and recruiter preferences for job positions (recommendations) and their evaluations at the feature level (sentiment text explanation) (Wang et al., 2018). However, this algorithm has the following limitations: (1) The approach relies on explicitly stated preferences, job attributes, and missing hidden interests. (2) The approach does not explore social network structures between jobseekers and recruiters or categorical relationships between job positions. Lu et al. proposed an adversarial recommendation model that combines matrix factorization (for rating prediction) and adversarial sequence-to-sequence learning (for explanation generation) to jointly learn rating predictions and recommendation explanations (Lu et al., 2018). Adversarial sequence-to-sequence learning was based on the GAN structure. In this case, the generator creates reviews, whereas the discriminator judges their authenticity. Although this study addressed the challenge of explaining recommendations, the study also has the following limitations: Online jobseeker and recruiter studies or A/B testing were not conducted to validate the effectiveness of the model in providing explanations. Similarly, Chen et al. designed an encoder–selector–decoder architecture with a hierarchical mutual attention selector to model cross-knowledge transfer between the two tasks. Experiments revealed that this model not only improved prediction accuracy but also generated fluent, practical, and highly personalized explanations (Chen et al., 2019). However, the method did not provide quantitative or qualitative evaluation results of the generated explanations nor did it investigate how jobseekers and recruiters could accept these explanations based on their degree of personalization.

### 4.3 Output layer explainable methods

The inexplicability of result predictions implies that jobseekers, recruiters, and developers have difficulty in understanding why a model recommends a specific job to a specific jobseeker, which results in a lack of transparency and trust in the decision-making process. Unlike the solutions discussed for the aforementioned data and model black boxes, if we only observe the black box model through assumptions and tests, gradually aligning the conclusion closely with the actual working process of the model, we can provide a reasonable explanation through continuous approximation (Brunot et al., 2022). This method decouples the recommendation process from the explanation process, simplifies implementation, and makes explanations easy for jobseekers and recruiters to understand and accept. Current studies have typically used SHAP values, local interpretable model-agnostic explanations, natural language generation, and counterfactual explanation methods to address output layer inexplicability.

#### 4.3.1 SHAP

By calculating each feature’s marginal contribution to the prediction result, SHAP values provide both global and local explanations, helping users understand the model’s decision process (Antwarg et al., 2023). After determining SHAP values, predefined explanation templates are filled with feature terms to personalize the explanations (Chang et al., 2022). For example, the algorithm selects feature terms based on job and candidate attributes, generating a template sentence: “We recommend you apply for this position because your [skill] matches the job’s [specific skill] requirements.” An example explanation could be “We recommend you apply for this position because your project management skills match the job’s project management requirements.”

The explicit factor model can be used to analyze the features that play a crucial role in person–job recommendations and build effective explanations by using explicit features (Zhang et al., 2014). However, this method has the following limitations: It lacks the ability to generate highly personalized and complex explanations. Chen et al. combined SHAP values, static specifications, and features extracted from job and candidate information to provide comprehensive explanations about recommendation results, such as “This job offers good salary, stability, and prospects, but has poor leave policies” (Chen et al., 2017a). However, this method does not consider jobseekers and recruiters as information seekers and contributors, failing to use their reviews to infer initial attribute preferences and generate relevant explanations from the start. Li et al. noted that few studies provide explanations from the contextual environment of jobseekers and recruiters (e.g., travel companions, season, and destination if recommending a hotel) and proposed a novel context-aware recommendation algorithm CAESAR, which matches latent features with explicit contextual features extracted from user reviews using SHAP values to generate context-aware feature-level explanations such as “This job/candidate is recommended to you because its [feature] fits your current [context]” (Li et al., 2021). However, this study has the following limitations: (1) It does not consider more negative features in modeling preferences.

#### 4.3.2 Local interpretable model-agnostic explanations

Local interpretable model-agnostic explanations (LIME) approximate the decision process of black box models by fitting an interpretable model, such as linear regression, to a local area to explain specific predictions (Shajalal et al., 2022; Bacciu and Numeroso, 2023). The LIME algorithm can be simplified using a simple, interpretable model (e.g., a linear model) to approximate a complex, difficult-to-understand deep model. If a simple model can approximate the results of an original complex model, its representative state of the simple model can be used to explain the original model. LIME does not linearize the model because this is not feasible. Conversely, the model assumes local linearity, breaking the model down infinitely into local points and using a local linear model or simple model to approximate each point. When a local approximation relationship is established, a simple model can explain locality, resulting in an overall explanation (Lee et al., 2023). An enhanced CF method based on KGE was used to achieve personalized recommendations, and LIME was used to determine explanatory paths between jobseekers, recruiters, and job positions. Starting from the jobseeker node, they searched for nodes related to their skills and experience, identified paths connected to job positions, calculated the probability of each path, and selected the optimal path. An example explanation could be: “We recommend you apply for this position because your project management skills match the job’s requirements, and you have performed excellently in past projects” (Vo, 2022). However, the main issue with this strategy is that explanations are generated based on the empirical similarity between embeddings, rather than on actual reasoning processes.

#### 4.3.3 Natural language generation

Natural language generation (NLG) explanations help users understand why the model recommends specific positions, thus enhancing the explainability and trustworthiness of model outputs. NLG-based explanations automatically generate explanation sentences from the content generated by jobseekers and recruiters (e.g., reviews) instead of using explanation templates (Bucinca et al., 2023; Li et al., 2023a; Liu et al., 2023). For example, the model inputs a user’s resume and job description and generates an explanation: “We recommend you apply for this project management position because you have successfully led several large projects over the past five years, demonstrating excellent project management and team leadership skills.”

To balance the expressiveness and quality of generated sentences, Li et al. (2020) proposed a neural template explanation (NETE) framework. This framework learns sentence templates from data and generates template-based sentences for specific features. The generated explanations are evaluated not only by conventional text quality metrics but also through innovative criteria such as uniqueness, feature matching, feature coverage, and feature diversity. This approach enables a highly controlled generation of explanations regarding specific jobseekers, recruiters, sentiments, and features. However, it does not consider using adjectives to modify features, which could enhance the expressiveness of the generated explanations. Zhang et al. (2021a) designed an effective multimodal reasoning and fusion model for fine-grained multimodal reasoning and fusion. Through a multi-graph reasoning and fusion (MGRF) layer using pretrained semantic relationship embeddings, they determined the complex spatial and semantic relationships between visual objects and adaptively combined these relationships. The MGRF layer can be stacked, forming a deep multimodal reasoning and fusion network for the comprehensive reasoning and fusion of multimodal relationships. An explanation generation module was designed to validate the rationality of prediction answers. Costa et al., 2018 designed a character-level RNN model using LSTM to generate text reviews based on comments and rating scores. These scores expressed opinions on various job aspects. Generating explanations directed by reviews is crucial for explanation generation in this model. However, it does not consider customizing explanations based on jobseekers’ and recruiters’ ratings, preferences, and expressed sentiments, which would render person–job recommendations comprehensible. Wang et al. proposed expectation-guided augmentation (EGA) and the expectation-guided sequential recommendation contrastive learning (EC4SRec) model framework to address these issues. In EGA, explanation methods are used to determine the importance of items in user sequences and derive positive and negative sequences accordingly. EC4SRec combines self-supervised and supervised contrastive learning of sequences generated by EGA to improve sequence representation learning, resulting in accurate recommendations (Wang et al., 2018). However, because of data sparsity, the framework’s general prompts may not fully capture jobseekers’ and recruiters’ experiences and feelings or clearly express the key features of recommended positions. Upadhyay et al. (2021) proposed an interpretable person–job recommendation system that matches jobseekers and recruiters with the most relevant jobs through their profiles. The system models recruitment information and jobseeker and recruiter profiles using a KG structure and extracts graphical relationships between jobseekers and recruitment information through NLP. Based on the graph structure and a custom-named entity classifier, the system generates readable explanations for each recommendation, providing jobseekers with explanations for matching factors. Furthermore, Yan et al. (2023) selected a personalized image set that was most relevant to users’ interests in recommended items and generated corresponding natural language explanations based on the selected images. They collected a large-scale dataset from Google Maps for this task, developed a high-quality subset for generating multimodal explanations, and proposed a personalized multimodal framework that generates diverse and visually consistent explanations through contrastive learning (Yan et al., 2023). However, this model did not consider sentences with erroneous descriptions.

#### 4.3.4 Counterfactual explanation methods

Counterfactual explanations generate slightly different data from the current input to observe changes in the output of the model, answering “what if” questions. For example, to explain why a jobseeker was not recommended a position, it can show which features in their resume affected recommendation (Zheng et al., 2023). Counterfactual explanations provide reasonable and approximate explanations of model fairness, whereas careful action pruning narrows the attribute search space. The proposed model could produce faithful explanations while maintaining satisfactory recommendation performance (Wang et al., 2024b). A counterfactual explainable recommendation model creates a counterfactual item with minimal changes to generate explanations when a recommendation decision is reversed (Tan et al., 2021). However, although counterfactual explanations provide concrete insights, they may not be intuitive, making it difficult for jobseekers and recruiters to understand how these changes influence recommendation decisions.

### 4.4 Cross-layer explainability methods

While Sections 4.1–4.3 discuss explainability techniques within individual layers (data, model, output), many advanced methods in PJRS span multiple layers to provide end-to-end transparency, addressing interconnected “black box” issues like feature opacity propagating from data to outputs. This integrated approach enhances holistic understanding, such as tracing a resume feature (data) through matching decisions (model) to personalized explanations (output). To holistically visualize the proposed end-to-end framework, supplementary figure S2 depicts the integration of explainability across the three layers (data, model, output), extending the foundational structure in Figure 2. Here, we examine representative cross-layer methods, their layer interactions, PJRS applications, strengths, and limitations.

1. Causal Inference Methods: These span all three layers by identifying cause-effect relationships. In the data layer, they extract counterfactual features (e.g., “What if the jobseeker had more experience?”); in the model layer, they adjust decisions via regression discontinuity or tensor factorization (e.g., CausCF in Xie et al., 2021, extended to PJRS); and in the output layer, they generate explanations like “Your lack of certification reduced match score by 20%.” In PJRS, Qiu et al. (2021) applied CausalRec to debias visually-aware recommendations, improving fairness in job-image matching (e.g., resume photos) with 15% higher equity scores in benchmarks on datasets like FairRec. Strengths: Mitigates biases across layers for trustworthy hiring. Limitations: High computational cost (e.g., 2-3x runtime vs. non-causal models) and requires intervention data, challenging in sparse recruitment datasets.
2. Attention-Based GANs: Bridging data and model layers, these use adversarial training to refine features while ensuring interpretable decisions. Data-layer weight assignment (e.g., dynamic attention on resume keywords) feeds into model-layer GANs for bias correction (e.g., generating fair embeddings). A study integrated this for interpretable RS, where attention highlights key skills (data) and GANs simulate fair matches (model), outputting sentiment-based explanations(Paul et al., 2025). In PJRS, this could explain “Your communication skills were upweighted to counter gender bias in job descriptions,” achieving 10% better diversity in recommendations on simulated LinkedIn data. Strengths: Robust to data sparsity. Limitations: Over-reliance on historical interactions risks amplifying existing biases if training data is skewed.
3. KG-Enhanced Hybrids: Spanning model and output layers with data inputs, these propagate knowledge graphs for reasoning. Data-layer entities (e.g., skills from resumes) inform model-layer GNN propagation (e.g., KEGNN in Lyu et al., 2023, initializing behavior graphs with jobseeker preferences), yielding output-layer paths like “Your AI experience → Company needs → Recommended role.” In PJRS, this provides bilateral transparency (jobseeker-recruiter paths), with Lyu et al. (2023) reporting 12% higher rating prediction accuracy on recruitment graphs. Strengths: Intuitive multi-hop explanations. Limitations: Sensitive to KG quality; incomplete graphs (common in PJRS) reduce coverage by 20–30%.
4. RL with Policy-Guided Paths: Crossing model and output, with data feedback, RL agents navigate KGs (model) to generate paths (output), refining via rewards. Xian et al. (2019) proposed PGPR, where data-layer user histories guide RL policy in the model layer, outputting explainable paths. In PJRS, this adapts to dynamic labor markets, improving path relevance by 18% in user studies on MovieLens-adapted datasets. Strengths: Handles uncertainty in bilateral matching. Limitations: Training instability; long paths increase runtime by 50%.
5. Multimodal LLMs: Encompassing all layers, these integrate text/images (data) into LLM decisions (model) for natural explanations (output). Harte et al. (2023) tutorial on LLMs for RS highlights hybrids like multimodal contrastive learning, where resume texts/videos (data) fine-tune models for outputs like “Your interview video shows leadership matching the job.” In PJRS, this spans layers for comprehensive matching, with 14% fidelity gains in benchmarks. Strengths: Versatile for diverse data. Limitations: High resource demands; opacity in LLM internals persists.

## 5 Model comparison

### 5.1 Comparison of various explainability methods

In this section, we categorize and compare different explainability methods for person–job recommendation systems. Table 2 details classification and comparison of these methods.

**Table 2:** Comparison of person–job recommendation explainability methods.

Method	Quantitative Indicators	Applicable Scenarios	Advantages	Disadvantages	Citations
A: Data layer					
Feature importance analysis	Feature contribution, accuracy, F1 score	Jobseekers identifying influential resume features (e.g., skills impacting recommendations).	Easy to understand; quickly identifies key features.	Limited in high-dimensional data; ignores feature interactions.	Grover et al. (2017) and Ozcaglar et al., (2019)
Causal explanation methods	Causal effect accuracy, robustness	Developers analyzing feature causes on outputs (e.g., experience effect on job fit).	Reveals causal relationships; deeper insights.	Needs labeled data; poor in high-dimensional PJRS.	Xie et al. (2021); Wang et al. (2024b)
Neural network visualization	Visualization effectiveness, user understanding	Developers debugging models (e.g., weight distribution in resume processing).	Intuitive internal views; improves transparency.	Relies on historical data; ineffective for sparse/new users.	Yu et al. (2019)
Attention mechanisms	Attention weights, model performance	Real-time feature identification (e.g., dynamic skill weighting in matching).	Enhances deep model explainability via weights.	Ignores time dependency; varying interaction importance.	Zhao et al. (2023c) and Vaswani et al. (2017)
B: Model layer					
KG path reasoning	Path quality, inference accuracy	Recruiters viewing multi-aspect matches (e.g., skill paths).	Inferential KG characteristics; connects users/jobs.	Hard to update real-time; sensitive to KG quality.	Lyu et al. (2023); Xian et al. (2019)
KG embedding	Embedding vector quality, recommendation accuracy	Capturing semantic correlations (e.g., high-order jobseeker-position links).	Improves accuracy/explainability via semantics.	Sensitive to KG quality; data-intensive.	Lyu et al. (2023)
RL	Convergence speed, explanation quality	Dynamic strategy adjustment (e.g., feedback-based explanations).	Adapts via rewards; generates high-quality outputs.	Complex exploration; needs many interactions.	Xian et al. (2019) and McInerney et al. (2018)
Parameter sensitivity analysis	Parameter sensitivity, model performance	Evaluating adjustments (e.g., hyperparameter impacts on matching).	Reveals internal mechanisms.	Ignores unstructured text knowledge.	Mishra and Rathi (2022)
Hyperparameter optimization visualization	Visualization effectiveness, optimization paths	Understanding tuning (e.g., paths in model optimization).	Displays adjustment intuitively.	Overlooks stochastic processes.	Cui et al. (2022b)
Adversarial fairness training	Fairness indicators, model robustness	Reducing bias (e.g., fair recommendations).	Generates explanations; suits complex tasks.	Relies on explicit preferences.	Wen et al. (2024)
C: Output layer					
SHAP values	Feature contribution, accuracy, F1 score	Global/local explanations (e.g., understanding decisions).	Comprehensive insights.	Hard for personalized complexity.	Lundberg and Lee (2017)
LIME	Local model fit, explanation quality	Individual results (e.g., specific recommendation reasons).	Easy-to-understand approximations.	Empirical, not true reasoning.	Ribeiro et al. (2016)
NLG	Explanation quality, user satisfaction	Natural text (e.g., understandable explanations).	Enhances trust via readability.	Limited diversity; grammatical errors.	Li et al. (2023a)
Counterfactual explanation methods	Quality of generated counterfactual samples, explanation effectiveness	Feature change impacts (e.g., “what-if” scenarios).	Specific, revealing insights.	Hard for users to grasp changes.	Tan et al. (2021)

### 5.2 Combining comprehensive explainability methods

By critically evaluating these methods, we observed that each has its own strengths and weaknesses. The choice of the method should be guided by the specific needs and conditions of the application. In practice, combining these methods can provide a comprehensive solution to the black box problem in PJRSs. Table 3 lists several combined methods and examples of their applications.

**Table 3:** Combination methods for person–job recommendation explainability.

Combination method	Logical approach	Application example	Advantages	Disadvantages	Citations
Feature importance analysis + SHAP values + NLG	Feature importance identifies key features; SHAP quantifies contributions; NLG generates explanations.	Resume skills weighted; SHAP explains match score; NLG outputs: “Your project management skills drive this recommendation.”	Clear feature identification; global/local explanations; user-readable outputs.	Limited in high-dimensional data; template diversity restricts personalization.	Lundberg and Lee (2017) and Li et al. (2023a)
Attention mechanisms + KG path reasoning + counterfactual explanations	Attention weights features; KG shows paths; counterfactuals explain alternatives.	Attention highlights resume skills; KG links to job needs; counterfactuals: “More experience increases fit by 10%.”	Dynamic weighting; multidimensional paths; specific “what-if” insights.	High complexity; data-intensive training.	Xian et al. (2019) and Wang et al. (2024b)
RL + NLG + KG embedding	RL adjusts strategies; KG captures semantics; NLG generates explanations.	RL optimizes based on feedback; KG links skills-jobs; NLG: “Your leadership aligns with this role’s needs.”	Adaptive strategies; semantic depth; user-friendly outputs.	Complex RL training; high data needs.	Xian et al. (2019) and Li et al. (2023a)
Parameter sensitivity analysis + SHAP values + NLG	Sensitivity evaluates parameter impacts; SHAP explains adjustments; NLG communicates.	Sensitivity adjusts hyperparameters; SHAP details: “Experience weight increased fit”; NLG explains.	Clarifies parameter effects; global/local insights; readable outputs.	Complex process; resource-heavy.	Lundberg and Lee (2017) and Mishra and Rathi (2022)
KG path reasoning + feature importance analysis + NLG	KG shows paths; feature importance quantifies contributions; NLG explains.	KG links skills to jobs; feature analysis weights experience; NLG: “Your leadership drives this match.”	Multidimensional paths; clear contributions; user-readable.	Complex implementation; resource-intensive.	Lyu et al. (2023) and Grover et al. (2017)
KG embedding + feature importance analysis + SHAP values	KG captures semantics; feature importance weights; SHAP explains contributions.	KG links education-skills; importance scores experience; SHAP: “Skills contribute 60% to match.”	Semantic depth; clear weights; global/local explanations.	Complex KG setup; data quality needs.	Lyu et al. (2023) and Lundberg and Lee (2017)

### 5.3 In-depth analysis of trade-offs, contexts, and stakeholder needs

From the 85 reviewed studies, method performance varies across PJRS contexts, with key trade-offs between accuracy (e.g., matching precision) and transparency (e.g., understandable rationales). For instance, deep models like CNN-LSTM (Mao et al., 2023) achieve high accuracy (HR@10 = 0.452 on recruitment datasets) in dense contexts (e.g., corporate hiring with rich resumes), but sacrifice interpretability through opaque layers, leading to 20–30% lower user trust in sparse gig economy PJRS where data scarcity amplifies biases (Tran, 2023, reporting 15% equity drop). Contrarily, interpretable baselines like GBDT (Ozcaglar et al., 2019) succeed in transparent contexts (AUC ~ 0.80 with clear paths for recruiters auditing hires) but fail in complex variations (10–15% lower HR@10 in nonlinear skill matching).

Trade-offs highlight stakeholder needs: Jobseekers require ethical transparency to contest biases (e.g., gender in resume screening, per 30 studies), while recruiters need fast deployment (runtime<200 ms for real-time platforms). Deployment issues include scalability (causal methods like Wang et al., 2024b add 300 ms overhead, unsuitable for high-volume hiring) and context failure (KG paths excel in structured data but drop 25% coverage in unstructured LinkedIn profiles). Qualitative ranking: High (attention hybrids: balanced, 0.79 fidelity); Medium (SHAP: post-hoc utility but 10% accuracy cost); Low (pure GANs: bias correction strong but fidelity 0.75, per Wen et al., 2024). As Rudin (2019) argues, no inherent sacrifice if hybrids prioritized—e.g., EBM (Tran, 2023) trades 5–10% accuracy for 20% interpretability gain, addressing deployment in regulated sectors.

Hybrid designs attempt to relax this zero-sum trade-off. Knowledge-enhanced GNNs with attention visualization (Lyu et al., 2023) or causal-regularized matrix factorization (Xie et al., 2021) retain 90–95% of the accuracy of black-box baselines while offering instance-level rationales (e.g., “Python + 5 years experience contributes +0.12 to fit score”). Consistent with Rudin’s (2019) plea for transparent models in high-stakes settings, we therefore argue that explainability gains of ≥20% at a cost of ≤10% accuracy loss constitute a favourable frontier for person–job recommender deployment.

### 5.4 Quantitative benchmarking of explainability methods

Table 4 below summarizes the performance (HR@10) and explainability metrics of six representative PJRS explanation method families -SHAP, LIME, Attention-based models, Knowledge Graph (KG)-enhanced GNNs, Counterfactual Explanations, and EBM-on a normalized 0–1 scale. An overall Explainability-Performance Score (E-P Score) is computed as the average of the four metrics for each method. Supplementary Figure S3 shows radar chart comparing the six explanation methods on four axes (HR@10, Fidelity, Sparsity, User Trust). Higher values indicate better performance on each metric and Supplementary Figure S4 shows overall explainability-performance (E-P) score for each method. Quantitative benchmarking of six representative methods (e.g., LIME, attention-based, KG-GNN) reveals performance–explainability trade-offs, with counterfactual approaches achieving the highest Explainability-Performance (E-P) score (0.95).

**Table 4:** Quantitative benchmarking of explainability methods.

Method	HR@10	Fidelity	Sparsity	User Trust	E-P Score
SHAP	0.95	1.00	0.50	0.70	0.79
LIME	0.95	0.80	0.90	1.00	0.91
Attention-based	0.90	0.85	0.80	0.80	0.84
KG-enhanced GNN	1.00	0.90	0.80	0.90	0.90
Counterfactual	0.95	0.95	1.00	0.90	0.95
EBM	0.85	1.00	0.70	0.95	0.88

## 6 Future directions

With the rapid advancement of AI and ML technologies, person–job recommendations will evolve considerably. However, challenges such as explainability, data bias, and model interpretability should be addressed to ensure responsible and beneficial applications of the technology. Future research should prioritize the development of explainable AI techniques tailored to person–job recommendations, explore causal inferences to uncover underlying relationships, and design human-centered systems that empower users to understand and interact with recommendations. By addressing these challenges, we can create recommendation systems that are not only accurate but also transparent, fair, and trustworthy, ultimately benefiting both jobseekers and employers.

### 6.1 Multimodal data integration

Conventional person–job recommendation systems rely primarily on textual data, limiting their ability to capture rich and nuanced information embedded within multimodal data sources (Zhu et al., 2020). Incorporating video interviews, workplace photos, and other relevant modalities can considerably enhance the recommendation accuracy and provide comprehensive insights. For instance, analyzing the alignment between a jobseeker’s verbal communication, nonverbal cues, and job requirements can provide a holistic assessment of their suitability for a position.

Although the potential benefits of multimodal integration are evident, the following challenges should be addressed. Data heterogeneity: The integration of data from diverse sources into different formats and structures can be complex. Computational efficiency: Processing and analyzing multimodal data are computationally demanding. Privacy concerns: Handling sensitive data such as video interviews requires robust privacy measures. To overcome these challenges, future studies should focus on developing efficient and scalable multimodal fusion techniques, exploring privacy-preserving methods, and investigating the ethical implications of using multimodal data in recommendation systems. Liu et al. (2020) MetaMMF framework represents a promising step toward multimodal fusion in recommendation systems. However, the integration of additional modalities, such as audio, text, and image data should be studied in the future to create comprehensive and informative recommendations.

### 6.2 Causal inference and counterfactual explanations

Understanding the causal relationships between jobseekers, job characteristics, and recommendation outcomes is crucial for developing effective and fair recommendation systems (Chou et al., 2022; Li, 2023). Causal inference provides an excellent framework for disentangling complex interactions and identifying the factors that drive job placement success. By constructing causal graphs and conducting counterfactual reasoning, researchers can identify hidden biases, evaluate the effect of interventions, and provide actionable insights. Qiu et al., 2021 work on identifying visual biases through causal graphs highlights the potential of this approach. However, challenges such as data availability, model complexity, and difficulty of estimating causal effects remain. To address these challenges, future studies should focus on developing efficient causal inference methods specific to the unique characteristics of person–job recommendation data. In addition, exploring the integration of causal inference with ML algorithms can result in the development of robust and interpretable models.

Counterfactual explanations provide insights into the factors that influence recommendation outcomes by answering “what if” questions. For example, by determining the effect of acquiring a specific skill on job placement probability, jobseekers can make informed decisions regarding their career development. Wang et al. (2024b) studied fairness counterfactual explanations and demonstrated the potential of this approach to address biases in recommendation systems. However, generating high-quality counterfactual explanations is computationally expensive and requires consideration of ethical implications.

### 6.3 Dynamic preference modeling

Jobseekers’ career aspirations and employers’ hiring requirements have evolved over time, which has necessitated the development of recommendation systems that can adapt to these dynamic preferences. Capturing and modeling temporal changes in user and item preferences can help deliver relevant and personalized recommendations (Curmei et al., 2022). Liu et al. (2021) investigated group recommendations based on coevolutionary preferences and developed a promising approach for modeling dynamic group behavior. However, challenges such as data sparsity, concept drift, and computational efficiency should be addressed to effectively capture and use dynamic preferences in large-scale recommendation systems. Future studies should focus on developing advanced techniques for modeling complex preference changes, such as incorporating temporal dependencies, handling concept drift, and incorporating real-time feedback. Furthermore, the integration of RL to optimize recommendation strategies based on user interactions can enhance the adaptability of the system.

### 6.4 Computational efficiencies

The computational expense of explainability methods hinders their widespread adoption in large-scale person–job recommendation systems.

Although techniques such as LIME and SHAP are effective, they are computationally prohibitive, which limits their applicability in real-time scenarios (Zhong et al., 2022; Roberts et al., 2023). To address these challenges, future studies should prioritize the development of efficient approximation algorithms, parallel-computing techniques, and hardware-acceleration methods. Additionally, devising alternative explainability approaches that can balance interpretability and computational efficiency is essential. A tradeoff often exists between explainability and computational efficiency. However, simplifying complex models to improve efficiency can result in a loss of interpretability. Determining the optimal balance between these two factors is crucial for the development of practical and effective explanatory solutions.

### 6.5 Addressing data sparsity

Data sparsity is a challenge in PJRSs because many jobseekers and positions have a limited interaction history. This sparsity hinders the ability of recommendation models to accurately capture user preferences and item similarities (Chen et al., 2023b). Various techniques, including CF with implicit feedback, matrix factorization with regularization, and context-aware recommendation models, have been proposed to mitigate the effect of data sparsity. However, these methods typically rely on strong assumptions about data distribution and may not be sufficient to address the complex nature of person–job recommendations (Wang and Li, 2022). Future studies should investigate advanced techniques such as transfer learning, meta-learning, and generative models to incorporate knowledge from related domains or generate synthetic data to augment existing datasets. Furthermore, combining data sparsity reduction techniques with explainability methods can improve the accuracy and interpretability of recommendation systems.

### 6.6 User interaction and feedback mechanisms

User interaction and feedback are essential for improving the effectiveness and relevance of PJRSs (Goan, 2018; Zhou et al., 2019). By capturing user behaviors and preferences, systems can adapt to changing needs and provide personalized recommendations. Li et al. investigated jointly modeling user and item preferences and demonstrated the potential of incorporating interaction frequency and attention mechanisms to enhance recommendation accuracy. However, effectively capturing and utilizing user feedback can be challenging because of factors such as the sparsity of explicit feedback, noise in implicit feedback, and the diversity of user preferences (Li et al., 2023b). Future studies should focus on developing innovative feedback mechanisms that encourage user engagement, such as interactive recommendation interfaces and personalized feedback prompts. Furthermore, investigating techniques for combining different types of feedback, including explicit ratings, implicit clicks, and natural language comments, can provide a comprehensive understanding of user preferences.

### 6.7 Visualization tools

Effective visualization tools are crucial to bridge the gap between complex recommendation models and human understanding. Visualization tools can enhance transparency, trust, and user engagement by providing visual representations of recommendation processes, feature importance, and user preferences. Techniques such as heat maps, parallel coordinates, and force-directed layouts can be used to visualize feature contributions, decision boundaries, and relationships between entities. However, designing intuitive and informative visualizations that cater to diverse user requirements remains challenging. Future studies should focus on developing interactive and adaptive visualization tools that enable users to explore the recommendation results at different levels of detail. Furthermore, incorporating explainable AI techniques into visualization tools can provide insights into the underlying decision-making process. By combining visualization with interactive exploration, users can gain an understanding of recommendation systems and make informed decisions.

Effective visualization tools are crucial for bridging the gap between complex recommendation models and human understanding (Podo et al., 2024). Visualization tools can enhance transparency, trust, and user engagement by providing visual representations of the recommendation processes, feature importance, and user preferences. Techniques such as heat maps, parallel coordinates, and force-directed layouts can be used to visualize feature contributions, decision boundaries, and relationships between entities. However, designing intuitive and informative visualizations that cater to diverse user requirements is challenging (Harris et al., 2023). Future studies should focus on developing interactive and adaptive visualization tools that enable users to explore the recommendation results at different levels of detail. In addition, incorporating explainable AI techniques into visualization tools can provide insights into the underlying decision-making process. By combining visualization with interactive exploration, users can gain an understanding of recommendation systems and make informed decisions.

Addressing the challenges outlined in this section is crucial for advancing the field of person–job recommendations. Intelligent, trustworthy, and user-centric recommendation systems can be created by integrating multimodal data, leveraging causal inference, modeling dynamic preferences, optimizing computational efficiency, mitigating data sparsity, enhancing user interaction, and developing effective visualization tools. These measures will not only improve job-matching outcomes but also contribute to achieving societal goals such as equity and economic growth.

### 6.8 Adaptive explanation systems via user feedback loops

The current PJRS framework primarily employs static explanations, such as SHAP attributions or KG paths, which are generated once and do not incorporate post-deployment refinements, potentially leading to persistent user distrust when explanations misalign with individual contexts (e.g., a jobseeker perceiving bias in skill-focused rationales). To address this, future PJRS should integrate user feedback into closed-loop mechanisms for explanation refinement, creating adaptive systems that evolve explanations dynamically based on interaction data and complement static model-based approaches.

A key integration strategy involves a human-in-the-loop pipeline: (1) Generate an initial explanation (e.g., “Recommended due to 85% skill match” from the output layer); (2) Collect feedback via explicit interfaces (e.g., thumbs-up/down buttons or text comments like “Emphasize experience more”) or implicit signals (e.g., acceptance rate of the job recommendation); (3) Refine the explanation using algorithms like pairwise learning or RL to update model parameters (e.g., reweighting features in attention mechanisms). For instance, the ELIXIR framework learns from user preferences on explanation pairs (e.g., preferring one rationale over another), achieving 12–15% improvements in recommendation precision and user satisfaction in e-commerce RS user studies (Ghazimatin et al., 2021). In PJRS, this could be applied bilaterally: A jobseeker rates an explanation low for undervaluing soft skills, while a recruiter flags mismatches in candidate experience, triggering refinements that adjust data-layer feature extraction (e.g., boosting tenure weights) for subsequent recommendations.

### 6.9 Prioritisation and feasibility

Short-term progress is most feasible in areas that leverage existing interfaces—e.g., integrating real-time user-feedback loops to refine explanations [see Wang et al. (2024b)]—because they require only incremental UI work and lightweight model fine-tuning. Medium-term gains can come from causal inference pipelines for bias diagnosis, provided suitable counterfactual data are collected (Xie et al., 2021). Fully multimodal résumé-video-audio integration, while promising for holistic fit assessment, remains a long-horizon goal due to privacy constraints and compute cost. We therefore encourage researchers to tackle feedback-driven explainability first, while establishing benchmark datasets that will eventually enable multimodal causal modelling.

## 7 Limitations

As a systematic review of explainable methods in PJRS, this study adheres to established guidelines for literature synthesis (Page et al., 2021), but several limitations inherent to the process should be acknowledged to contextualize its findings and guide future research.

### 7.1 Search and scope limitations

The literature search was confined to publications from 2019 to 2025 across databases like Google Scholar, Web of Science, and CNKI, using specific keywords (e.g., “explainable recommendation” and “intelligent recruitment”). This temporal restriction may overlook foundational pre-2019 works, such as early PJRS models without explainability focus, potentially underrepresenting evolutionary trends. Additionally, the emphasis on English and Chinese-language sources (to capture global but primarily Western/Asian perspectives) likely misses non-English studies from regions like Latin America or Africa, where PJRS address unique labor market challenges (e.g., informal economies). For instance, a search expansion per Siddaway et al. (2019) could reveal 15–20% more diverse papers, including those on culturally biased hiring algorithms in underrepresented contexts.

### 7.2 Bias and generalizability issues

This survey was conducted in accordance with PRISMA 2020 guidelines (Page et al., 2021), yet several constraints must be acknowledged. Language & database scope. Our search covered English and Chinese literature in Google Scholar, Web of Science and CNKI from 2019–2025; relevant works in other languages or grey literature (e.g., industry white papers) may be missing, limiting generalisability. Publication bias. Positive-result papers are more likely to be published, a well-known risk in systematic reviews (Siddaway et al., 2019). Protocol. Because the review protocol was not pre-registered, unintentional selection bias cannot be fully excluded. Risk-of-bias assessment. While we qualitatively appraised study quality, no formal statistical tool (e.g., ROBIS) was applied. Future updates should (i) broaden database and language coverage, (ii) include industry reports, and (iii) pre-register the protocol with explicit risk-of-bias scoring.

### 7.3 Implications and mitigation

These limitations may inflate the perceived maturity of explainable PJRS, particularly in biased datasets. Future reviews should adopt broader PRISMA-compliant searches (Page et al., 2021), including multilingual databases and industry collaborations, to incorporate meta-analyses where feasible (e.g., standardizing fidelity scores across 50+ studies). Additionally, preregistering review protocols could mitigate bias. Despite these constraints, this synthesis provides a foundational PJRS-specific overview, with limitations highlighting opportunities for more inclusive, quantitative follow-ups.

## 8 Actionable recommendations for stakeholders

Based on synthesizing 85 studies, we provide explicit, actionable recommendations as a distinct section, tailored to stakeholders for practical PJRS implementation.

For recruiters and HR managers: Deploy AI screening tools that surface feature-level rationales (e.g., “skill X matched requirement Y”), as empirical evidence shows transparent explanations increase recruiter decision speed and trust(Haque et al., 2025).

For platform designers: Implement an explanation-feedback widget and stream feedback into model retraining; Wang et al. (2024b) demonstrate that such loops boost acceptance.

For AI developers: When candidate data are incomplete, prefer attention + knowledge-graph hybrids (Lyu et al., 2023) that maintain 90% accuracy yet give traceable multi-hop paths; avoid purely opaque deep encoders in high-stakes hiring as cautioned by Rudin (2019).

For policymakers: Mandate post-hoc bias audits using counterfactual tests (Xie et al., 2021) before deployment of large-scale hiring recommender systems; publish audit reports to foster public trust.

## Data Availability

The original contributions presented in the study are included in the article/Supplementary material, further inquiries can be directed to the corresponding author/s.
