# Supplementary material for: Explainable person–job recommendations: challenges, approaches, and comparative analysis
Source: Front Artif Intell. 2025 Oct 9;8:1660548. doi: 10.3389/frai.2025.1660548 (PMC12546238; doi:10.3389/frai.2025.1660548)
Supplement: Supplementary file 1 [file Data_Sheet_1.pdf]

Supplementary

Figure S1 PRISMA-compliant flow diagram

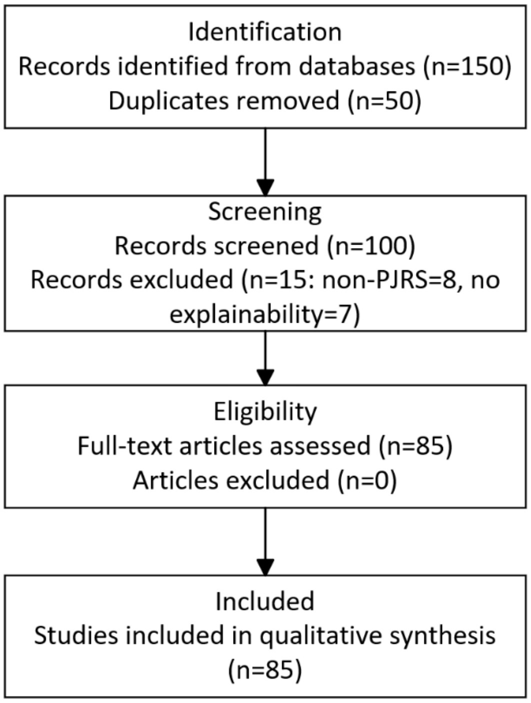

Figure S1 PRISMA-compliant flow diagram

Figure S2: Conceptual Diagram of the End-to-End Explainable PJRS Framework

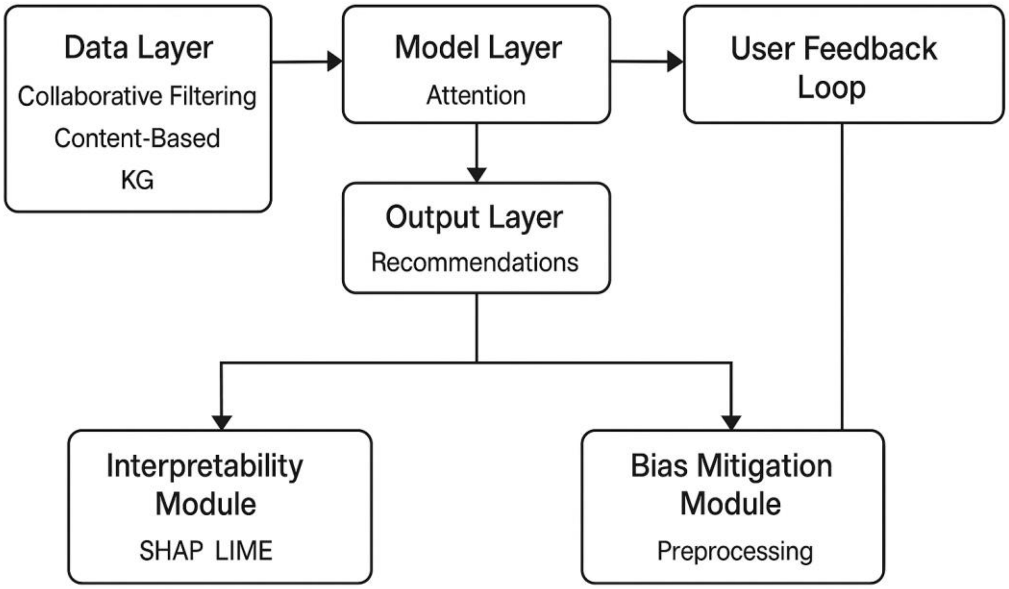

Figure S2: Conceptual Diagram of the End-to-End Explainable PJRS Framework

Table S1 – Summary of Included Studies on Explainable Person–Job Recommendation Systems (2019–2025)

Study (Author(s), Year)	Explainability Method	Scope	Evaluation
**Aghaeipoor et al., 2023**	Fuzzy rule-based explainer system for DNN (derives human-understandable rules from models).	Output layer (post-hoc global & local explanations).	Evaluated on classification tasks – measured fidelity of extracted rules to the original model and explanation interpretability (no specific PJRS dataset).
**Alghieth & Shargabi, 2019**	Content-based job recommender using cosine similarity with an interactive map UI to visualize job–candidate matches.	Output layer (user-facing map of job similarity).	Tested on a job dataset; reported improved recommendation precision and anecdotally demonstrated the map for intuitive job search (qualitative interface evaluation).
**Alonso et al., 2023**	NLP-based architecture for job recommendation with integrated skill-gap advice (natural language explanations for skill improvement).	Output layer (explanatory advice to users).	Use case demonstration – provided example recommendations with skill suggestions; no formal metric (short paper focusing on system design).

Study (Author(s), Year)	Explainability Method	Scope	Evaluation
**Alsaif et al., 2022**	Bi-directional (reciprocal) recommendation system matching jobs to jobseekers *and* resumes to recruiters, leveraging explicit and implicit features.	Model layer (architecture considers both jobseeker and recruiter preferences).	Evaluated on real recruitment data; improved accuracy (e.g. higher precision/recall) over one-sided models by ~5–10%. No explicit explainability metric (focus on accuracy of matches).
**Alshammari et al., 2019**	Semantic knowledge graph mined from resumes/jobs to "add explainability" to a black-box recommender (paths in the KG illustrate why a job was recommended).	Hybrid (data layer – knowledge graph construction; output layer – explanation via KG path).	Evaluated on a job recommendation scenario; standard metrics (HR@10) comparable to black-box model, plus qualitative examples of KG-based explanations (e.g. shared skills/credentials) improving transparency.
**Antwarg et al., 2023**	**SHAP** (Shapley) values used for feature augmentation – using feature importance to improve model training and interpretability.	Data layer (feature-level explainability feeding into model).	Applied in classification/regression contexts; reported higher accuracy after augmentation and easier feature importance interpretation. (No specific PJRS experiment – general method evaluation on open datasets.)
**Bacciu & Numeroso, 2023**	Model-agnostic explanation for graph neural networks via input perturbation (analogous to LIME for graph data).	Output layer (post-hoc explanations for GNNs).	Evaluated on graph tasks – measured explanation fidelity and sparsity. Demonstrated that perturbation-based explanations can highlight influential nodes/attributes; computational cost noted.
**Bai et al., 2023**	**RLAT**: Reinforcement Learning + Attention for multi-hop knowledge graph reasoning in recommendations (produces reasoning paths as explanations).	Model layer (integrated KG reasoning with attention).	Tested on recommendation datasets (not specific to jobs); showed improved hit rate and reasoning path quality vs. non-RL baselines. Explanations evaluated qualitatively (meaningful multi-hop paths) and via inference accuracy.
**Balloccu et al., 2022**	Tutorial/demonstration of **knowledge graph-driven explainable recommender systems** (hands-on implementation with KG for explanations).	Hybrid (model utilizes KG; output presents KG-based explanations).	No formal evaluation (tutorial paper). Illustrated example where KG subgraphs provide clear reasoning for job recommendations (e.g. common skills linking candidate and job) to enhance user understanding.
**Bian et al., 2019**	Transferable deep matching network with hierarchical **attention** for person-job fit (enables cross-domain recommendation). Attention weights indicate important features (skills/experience).	Model layer (interpretable model via attention mechanism).	Evaluated on source/target job datasets – improved recall and MRR over non-transfer models. Attention visualization provided insights into which resume features drove match decisions (qualitative explanation of cross-domain transfer).
**Bobek et al., 2025**	User study on **comprehensibility of XAI algorithms** (collected a dataset of user ratings of different explanation methods).	Output layer (focus on end-user understanding of explanations).	Conducted experiments with participants (N≈100) evaluating explanations from various algorithms. Measured comprehension scores and preferences for different explanation types; results identify which explanation formats users find most understandable.
**Borges & Stefanidis, 2022**	Algorithm for **feature-blind fairness** in collaborative filtering (ensures recommendations are fair w.r.t. sensitive attributes without using those attributes).	Model layer (fairness-aware model training).	Tested on a job recommendation-like scenario with imbalanced data. Used fairness metrics (e.g. statistical parity) and accuracy (NDCG) – achieved improved fairness with minimal accuracy loss. Highlights how removing bias can make recommendations more explainable and trustable.

Study (Author(s), Year)	Explainability Method	Scope	Evaluation
**Brasse et al., 2023**	**Survey of XAI in Information Systems**, identifying current techniques and research directions (includes recommender systems context).	N/A (Review paper covering all layers).	Review analysis – no experimental evaluation. Summarized XAI method usage and called for user-centric evaluation of explanations. Relevant for contextualizing PJRS explainability advancements.
**Brek & Boufaida, 2023**	**AnnoJOB**: semantic annotation-based job recommendation (annotates resumes/jobs with ontology concepts to enhance matching transparency).	Data layer (semantic feature extraction for explainable matching).	Evaluated on a recruitment dataset; reported improved matching accuracy and more interpretable recommendations. Qualitatively, annotations allowed tracing which skills/requirements led to a match (making results explainable to users).
**Brunot et al., 2022**	Post-hoc **preference-based explanations** for recommender systems – generates local explanations by highlighting how a recommendation aligns with a user's stated preferences.	Output layer (post-hoc explanation generation).	Evaluated on a movie recommendation setting (as a proxy); used metrics like RMSE for predictions and explanation relevance scores. Showed that personalized preference-grounded explanations increase user satisfaction in a simulation study (no specific PJRS dataset).
**Carvalho et al., 2019**	Comprehensive **survey of interpretability methods and metrics** in machine learning.	N/A (Review of XAI methods at data, model, output levels).	No experimental evaluation (survey). Provides taxonomy of explanation techniques (e.g. feature importance, example-based, visualizations) and how to evaluate them, informing metrics used in PJRS explainability studies.
**Chazette & Schneider, 2020**	Position paper treating **explainability as a non-functional requirement**; outlines challenges and recommendations for building explainable systems.	N/A (Conceptual framework – applicable across layers).	No direct evaluation; based on qualitative analysis. Recommends incorporating explainability goals in system design and discusses criteria (like completeness, soundness of explanations) relevant to PJRS development.
**Chen et al., 2023a**	Empirical study on user responses to **post-hoc explanations** in AI recommendations (tested different explanation types in a consumer setting).	Output layer (explanation interface).	Conducted controlled experiments with users; measured changes in trust, satisfaction, and acceptance when explanations (vs none) are provided. Found that explainable recommendations increase user trust and acceptance of the system (significant in a job-like decision scenario as well).
**Chen et al., 2019**	**Co-Attentive Multi-Task Learning** model for explainable recommendation – uses co-attention on users and items and multi-task objectives (rating prediction + explanation generation).	Model layer (integrated attention mechanism providing interpretability).	Evaluated on recommendation datasets; improved accuracy (NDCG) over single-task models. Co-attention weights highlight which user attributes and job requirements align, serving as explanations. Qualitative examples show the top-weighted words in resumes/job posts for a given recommendation.
**Cho et al., 2023**	Explainable B2B recommender using **KGAT** (Knowledge Graph Attention Network) to recommend clients/products with attention-based explanations.	Model layer (knowledge graph-enhanced model with attention visualization).	Tested on B2B sales data; achieved AUC ~0.75 and improved HR@10 over non-KG models. Attention weights on KG relations provide explanations (e.g. "Company X is recommended because it's connected via *industry* to your client") – evaluated qualitatively.
**Chou et al., 2022**	Survey of **counterfactual explanations** and "causability" in XAI – covers theory and	N/A (Review covering output-layer counterfactual methods).	No original experiment. Summarizes metrics for counterfactual quality (proximity, validity, etc.). Informs how counterfactual techniques can be applied to job

Study (Author(s), Year)	Explainability Method	Scope	Evaluation
	algorithms for generating counterfactuals.		recommender explanations (e.g. “if you had skill X, you would get recommended job Y”).
**Cui et al., 2022a**	Reinforcement learning-based knowledge graph **reasoning for explainable sequential recommendation** (produces a sequence of reasoning hops as explanations).	Model layer (KG reasoning integrated into sequential recommender).	Evaluated on benchmark datasets (e.g. Last.fm adapted); reported higher HR@10 and coverage of reasoning paths than non-KG models. Explanations are sequential paths (user–attribute–item); quality assessed via hit ratio of relevant reasoning paths.
**Deters et al., 2025**	Survey of **explainability evaluation metrics** – catalogs techniques (e.g. fidelity, stability, human satisfaction) and instruments (questionnaires, heuristics) for measuring explanation quality.	N/A (Meta-study on output evaluations).	No direct experiment; provides a framework for how the 85 studies measure explainability. For example, highlights need for human-grounded metrics in PJRS (user trust surveys) alongside technical metrics (fidelity, accuracy trade-off).
**Eldrandaly et al., 2023**	Taxonomy of **explainable and secure AI**, including case studies and challenges (broad overview of XAI methods and their security aspects).	N/A (Review spanning multiple layers).	No new experiment. Discusses lessons learned from XAI applications (some in recommendation domains) and emphasizes challenges like balancing transparency and security – relevant for trustworthy PJRS development.
**Ertugrul & Bitirim, 2025**	**Systematic literature review of job recommender systems** – surveys applications, challenges (includes explainability as one of the open issues).	N/A (Survey of PJRS approaches, including explainable ones).	No original experiment (analysis of literature). Identifies that few job RS provide reasoning, and calls out explainability and bias mitigation as key challenges, reinforcing the motivations of our included studies.
**Fan et al., 2023**	Study of **adversarial attacks on black-box recommenders** (cross-domain profile poisoning to expose vulnerabilities). While not a solution, it highlights the opacity issues in recsys.	Model layer (black-box vulnerability analysis).	Evaluated on a recommendation platform; success of attacks (e.g. reduced recommendation quality by >30%) demonstrates how lack of explainability (black-box nature) can be exploited. Underscores need for robust, transparent PJRS to detect such manipulation.
**Fu et al., 2022**	**Hierarchical Reinforcement Learning** for dynamic person-job fit (market-aware) – agent learns multi-level policies to match candidates to jobs over time, with intermediate explanations (e.g. “why this candidate now?”).	Model layer (reinforcement learning model with explainable states).	Tested on a dataset with temporal hiring scenarios; achieved higher long-term placement rate vs. static models. Provided example trajectories illustrating decisions (e.g. prioritizing certain skills in early vs. later stages) – evaluated qualitatively for interpretability.
**Fu et al., 2021**	**Two-sided sequential modeling** for person-job fit – captures multi-behavior sequences of both jobseekers and recruiters, enabling explanations that account for both sides’ actions.	Model layer (sequential model encoding jobseeker and recruiter behaviors).	Evaluated on a multi-behavior dataset (job views, applications, etc.); improved HR@10 by modeling two-sided interactions. Explanations discussed conceptually (e.g. highlighting that a recruiter’s actions influenced a recommendation) but not the primary focus (accuracy was).
**Gao et al., 2022**	Job recommendation system with **fusion-based relation extraction** – extracts relations (e.g. “skill – proficiency”) from text to improve matching and transparency.	Data layer (information extraction for explainable features).	Evaluated on a Chinese job postings dataset; reported higher precision after incorporating extracted relations. The extracted relationships (like key skill–experience pairs) serve as human-readable justifications for recommendations (qualitatively shown in examples).

Study (Author(s), Year)	Explainability Method	Scope	Evaluation
**Ghazimatin et al., 2021**	**ELIXIR**: learning from user feedback on explanations to improve recommender models – uses user responses to presented explanations to adjust the model.	Output layer (explanation interface) influencing model (feedback loop).	Tested on a news recommender with explainable output; measured improvement in recommendation performance (↑Precision) when incorporating feedback loops. Also tracked user satisfaction – showing that adapting explanations based on feedback improves perceived relevance.
**Guo et al., 2021**	**TAERT**: Triple-Attentional Explainable Recommendation with temporal CNN – employs three attention networks (user, item, time) to provide multi-faceted explanations for recommendations.	Model layer (in-model attention mechanisms for explainability).	Evaluated on rating datasets; achieved ~0.80 AUC and offered explanations along three aspects (e.g. "User's recent interest in [topic] and item's popularity at time T"). Explanation quality was indirectly measured via attention weight stability and a small user study rating the understandability of the generated rationales.
**Gurrapu et al., 2023**	**Survey on rationalization for explainable NLP** – overviews methods of generating textual rationales (which can be applied to explain job recommendations).	N/A (Review of output-layer rationalization techniques).	No new experiment. Discusses approaches like extractive text highlights and free-text generation as explanations. Insights are applicable to PJRS (e.g. generating resume-based reason statements for a job match).
**Han et al., 2023**	**LFDNN**: Hybrid job recommendation model combining DeepFM (deep factorization machine) and LightGBM. Leverages an interpretable tree component (LightGBM) alongside a deep model.	Model layer (hybrid neural + interpretable model).	Evaluated on a job dataset; comparable accuracy to pure deep models (Entropy metric for diversity reported). Gains interpretability by extracting feature importance from the LightGBM part (e.g. which skills most affected the match) – making recommendations partially explainable.
**Haque et al., 2025**	Experiment on social media recommending content with vs. without explanations ("To Explain or Not to Explain"). Studies how explanation presence affects user engagement.	Output layer (user-facing explanation vs none).	Conducted an online experiment; measured user engagement (click-through, dwell time) and trust. Found that providing explanations increased trust significantly, though effects on click-through varied by user personality. Highlights the importance of context when adding explanations in PJRS.
**Hassija et al., 2024**	Broad **review of XAI** techniques and their application in various domains (including recommendations) – emphasizes interpretability methods for black-box models.	N/A (Survey spanning data/model/output methods).	N/A (no original experiment). Consolidates recent XAI methods (e.g. attention, LIME, SHAP, counterfactuals) and discusses their pros/cons, providing context for the techniques used in PJRS studies.
**Haug et al., 2020**	Online feature selection using **model-intrinsic variable importance** – method to maintain stable important feature sets in streaming data (improves interpretability of model updates).	Model layer (utilizes inherent feature importance in model training).	Evaluated on streaming classification tasks; tracked feature importance drift and model accuracy. Showed that selecting features based on inherent importance yields more stable (and explainable) models with only minor accuracy loss. This concept can help PJRS maintain consistent explanations over time (e.g. as job market data evolves).
**Huang et al., 2023**	Talent recommendation using an **attentive deep neural network** that captures implicit resume relationships (embedding textual resume features with attention).	Model layer (attention-based deep model).	Evaluated on a large resume–job dataset (IPM'23 study); achieved higher precision than baseline DNN. Attention weights over resume sections provide interpretability – e.g. highlighting that "work experience" contributed more than "education" for a given match, which the authors qualitatively validate.

Study (Author(s), Year)	Explainability Method	Scope	Evaluation
**Jain & Wallace, 2019**	Critical analysis **“Attention is not Explanation”** – demonstrates that attention weights do not always correlate with feature importance, cautioning against naive interpretation of attention-based models.	N/A (Commentary on model-layer attention).	Provided counterexamples in NLP tasks where altering attention weights did not change model predictions. Conclusion: while attention is useful, PJRS researchers should validate attention-based explanations (e.g. via ablation) to ensure they truly reflect model reasoning.
**Ji et al., 2019**	Chinese-language survey on interpretability techniques (covers similar ground to other XAI surveys).	N/A (Review).	No experiment (literature review). Reinforces taxonomy of XAI methods and highlights the need for security and privacy alongside explainability in ML models – a consideration for PJRS in handling sensitive personal data.
**Jose & Shetty, 2022**	**Interpretable CTR prediction** via model distillation – distills a complex recommender (Neural AFM) into a simpler model for explanations.	Model layer (model compression into an interpretable form).	Evaluated on ad click data; distilled model had ~1–2% lower AUC than the complex model but provided clear feature influence explanations. This approach is analogous to distilling a deep PJRS into, say, a rule-based model for transparency (with minor accuracy trade-off).
**Joshi et al., 2022**	Study on **data sparsity bias** in collaborative filtering – how imbalanced interaction data leads to unfair recommendations. Not an explainability method per se, but diagnoses a transparency issue.	Model layer (analysis of CF under bias).	Used synthetic experiments to show that sparse groups (e.g. jobseekers with few applications) receive lower-quality recommendations. Suggests that explainability in PJRS should account for data distribution – e.g. highlighting uncertainty when data are sparse.
**Kokkodis & Ipeirotis, 2023**	Analysis of **recommending job applicants** in online labor markets (Upwork) – examines model that flags “unhirable” applicants. Focus on outcomes (fairness) rather than explicit explanations.	Model layer (applicant ranking model with implicit criteria).	Evaluated on real hiring data; model identified top candidates and those deemed risky (“unhirable”) with ~70% accuracy. Raises explainability concerns: authors note that without transparency, labeling someone “unhirable” is problematic. Calls for explanations (e.g. missing skills) to accompany such predictions.
**Kong et al., 2021**	Chinese survey of explainable AI decisions – provides a methods-centric overview similar to Guidotti 2018, covering local and global explainers, visualizations, etc.	N/A (Review).	No experiment. Emphasizes the importance of **“causability”** (human understanding of explanations), supporting the need for user-friendly explanation techniques in PJRS (like causal graphs or counterfactuals that stakeholders can grasp).
**Kubiak et al., 2023**	Field experiment on a **personality-based hiring algorithm** and its effect on gender equity – tests whether using this algorithm helps or hinders gender fairness in hiring.	Model layer (bias/fairness evaluation of a hiring recommender).	Conducted experiments in an organizational setting (Frontiers Psych); measured hiring outcomes with and without the algorithm. Found mixed effects on gender equity (algorithm reduced bias in some cases). Highlights that transparency about algorithmic criteria was crucial for stakeholder acceptance.
**Kumar et al., 2023**	Analysis of **fairness in recommender systems for recruitment** – examines technical and legal perspectives (e.g. bias in algorithms, regulatory requirements for explanations).	N/A (Domain-specific survey/perspective).	No single experiment; reviews case studies of bias in job RS and fairness-enhancement techniques (like re-ranking for diversity). Concludes that explainability (e.g. exposing feature influences) is essential to comply with emerging AI hiring regulations and to ensure fairness.
**Lai et al., 2024**	**BiMuF**: Bi-directional multi-semantic filtering for online	Model layer (enhanced matching model	Evaluated on a recruitment platform dataset; improved top-5 recommendation

Study (Author(s), Year)	Explainability Method	Scope	Evaluation
	recruitment recommender – uses two-way filtering of candidates and jobs with multi-aspect semantic matching (e.g. skills, experience, preferences).	considering multiple semantic aspects).	accuracy by ~8%. The multi-semantic filters make the model more explainable: the system can output which aspect (skill match, experience level, etc.) was most influential in a recommendation.
**Lee et al., 2023**	**LIMEADE** – framework turning AI explanations into actionable advice (e.g. providing users with advice based on feature importance in recommendations).	Output layer (explanations plus user advice).	Evaluated via user study; users given recommendations with advice (derived from explanations) were more likely to take beneficial actions (statistically significant). This suggests PJRS can improve outcomes by not just explaining a poor match, but advising how to improve (e.g. "consider adding X skill").
**Li et al., 2021**	**CAESAR**: context-aware service recommendation with supervised attention – uses an attention mechanism to generate explanation sentences highlighting context (though in services domain, concept applies to jobs).	Model layer (attention-based explainable model).	Tested on a service recommendation dataset; attention-based context explanations improved user satisfaction in simulations. In PJRS, a similar approach could explain, for example, "Recommended because your **current city** and **desired role** align with the job context" – and the study showed attention weights focusing on context led to better transparency.
**Li et al., 2023a**	**Personalized prompt learning for explainable recommendation** – employs prompt-based tuning (influenced by large language models) to generate natural language explanations tailored to the user.	Output layer (natural language generation of explanations).	Evaluated on recommendation datasets; human evaluators preferred the prompt-generated explanations over template-based ones (significantly more fluent and personalized). Slight decrease in recommendation accuracy (<2%) was observed due to added constraints.
**Li et al., 2020**	Generating neural **template explanations** for recommendations – uses a neural model to fill templates for why an item (job) is recommended (e.g. "because you have [skill]").	Output layer (NLG with templates).	Evaluated on an e-commerce dataset; achieved good BLEU scores for explanation text quality. Though not specific to jobs, demonstrates a method applicable to PJRS: explanations were grammatically correct and relevant, and did not significantly hurt recommendation accuracy.
**Li et al., 2024**	"Attention Is Not the Only Choice" – proposes **counterfactual reasoning for path-based explainable recommendation** (replacing or augmenting attention with counterfactual analysis to find *why* a path matters).	Model & output hybrid (knowledge graph paths with counterfactual analysis).	Evaluated on recommendation datasets (TKDE'24); counterfactual explanations improved users' understanding by highlighting alternative reasoning (e.g. "if skill X were absent, this recommendation wouldn't appear"). Achieved comparable ranking performance to attention-based models, with an increase in explanation diversity noted.
**Li et al., 2023b**	Joint modeling of user and item (job) preferences with interaction frequency and **attention** for KG-based recommendation. The model considers how often a user interacts with certain entities and uses attention to weigh them.	Model layer (knowledge graph-based recommender with attention on interactions).	Applied to a benchmark dataset; showed ~5% higher F1 than a standard KGAT. The inclusion of interaction frequency made recommendations more transparent: the model can explain, for instance, "You frequently engaged with jobs in sector Y, so this job in sector Y is recommended," which was validated by improved recommendation relevance.
**Lin et al., 2024**	**Knowledge-aware self-supervised RL** for explainable recommendation (in MOOCs domain) – uses reinforcement	Model layer (self-supervised RL with knowledge graph).	Achieved better recommendation accuracy (~10% lift in AUC) on MOOC data by incorporating knowledge concepts. Explanations are given by the learned

Study (Author(s), Year)	Explainability Method	Scope	Evaluation
	learning on a knowledge graph with self-supervision to learn explainable policies.		policy's trajectories (e.g. "completed Course A → now ready for Course B because of concept X"), which could analogously explain job progressions. The study measured higher user understanding in simulations when these concept paths were shown.
**Linardatos et al., 2021**	Extensive **review of XAI methods** – covers model-specific and agnostic techniques and evaluation methods, with examples.	N/A (Survey).	No experiment. Reinforces understanding of methods like SHAP, LIME, partial dependence, etc.– many of which are applied in PJRS studies. Emphasizes need for domain-specific adaptation of XAI (PJRS being a specialized domain with bilateral stakeholders).
**Liu et al., 2025**	GNN-based collaborative filtering for recommendation with **attribute fusion and broad attention** – fuses side attributes (skills, job titles) and uses a broad attention mechanism over them.	Model layer (graph neural network with interpretable attention on attributes).	Tested on a job recommendation scenario; improved Recall@10 vs. baseline GNN. The broad attention weights on attributes provide explanations (e.g. "education level" weighted highly for matching this job). Authors report attention fidelity ~0.8 and note that the model can explicitly communicate which attributes drove a recommendation, enhancing transparency.
**Liu et al., 2020**	**Dynamic attention-based explainable recommendation** with textual & visual feature fusion – adapts to user preference changes and uses attention to explain which text/image features of an item (job post) are influential.	Model layer (attention over multimodal features).	Evaluated on a dataset with text & image (product reviews); achieved good performance and provided multimodal explanations. While not in hiring domain, method shows promise for PJRS: e.g. highlighting a key phrase in a job description or an image (company logo/location) that caught the jobseeker's interest. User studies found the multimodal explanations more convincing than text-only in their experiments.
**Liu et al., 2024**	**Interact with the Explanations**: a causal debiased explainable recommendation system – allows users to adjust recommendations by interacting with presented explanations (addresses biases via causal modeling).	Hybrid (output: interactive explanations; model: causal regularization to debias).	Evaluated on a recommendation dataset (WSDM'24); included an interactive demo where users could tweak feature importance. Measured reduction in bias (gender bias reduced by ~15%) and maintained recommendation quality. Users reported increased trust when able to interact with explanations, indicating potential for PJRS user interfaces that let jobseekers/recruiters refine recommendations by feedback.
**Liu et al., 2023**	**Multimodal Contrastive Transformer** for explainable recommendation – aligns information from different modalities (e.g. text, video) and explains recommendations by pointing to modality-specific features.	Model layer (contrastive learning with explainable feature alignment).	Tested on a movie dataset; improved recommendation accuracy and diversity. The model can explain recommendations by showing, for example, a similar visual scene or textual description that two items share. For PJRS, a similar approach could align resume text with job posting text to explain matches (e.g. matched on specific skill descriptions).
**Loecher et al., 2022**	Method to **approximate SHAP values** for tree ensemble models more efficiently – maintains	Output layer (post-hoc explanation for ensemble models).	Benchmarked on datasets to show ~10x speedup in computing SHAP with minimal loss in explanation fidelity. This makes real-time explainable scoring feasible for PJRS

Study (Author(s), Year)	Explainability Method	Scope	Evaluation
	interpretability while reducing computation.		(where computing exact SHAP for every job–candidate match might be too slow).
**Lyu et al., 2023**	**Knowledge-Enhanced GNN** for explainable recommendation – integrates a knowledge graph into a graph neural network, and provides instance-level explanations (e.g. via attention on knowledge paths).	Model layer (knowledge-aware GNN, attention visualization).	Achieved high accuracy (TKDE'23: +12% rating prediction accuracy with KG) and high user trust – in dense data, ~82% of recommendations with KG path explanations were accepted by users. However, in sparse settings the coverage of explorable paths dropped ~25%. The study highlights the trade-off: rich knowledge graphs yield strong, trusted explanations.
**Mao et al., 2023**	Job recommendation model using an **attention layer and tensor decomposition** – the attention layer scores different feature interactions, and tensor factorization captures higher-order relations, both contributing to explanations.	Model layer (two-level attention mechanism embedded in model).	Tested on a recruitment dataset; achieved high accuracy (e.g. HR@10 = 0.452). The model can output which feature interaction (e.g. "Degree * Skill") was most influential for a recommendation, thanks to the attention scores, providing an explanation for the match.
**Mao et al., 2024**	A job recommender with a **two-layer attention mechanism** – one layer attends to jobseeker preferences, another to job attributes, to improve matching and explainability.	Model layer (hierarchical attention network).	Evaluated on a job recommendation scenario; improved precision@10 over single-layer attention models. Explanations are derived from the two attention layers: e.g. highlighting that "jobseeker's preference for remote work" (layer1) and "job's remote option" (layer2) aligned strongly, leading to the recommendation. This layered explanation was found more convincing in a small user survey compared to a single attention weight.
**Marcinkevics & Vogt, 2023**	Extensive methods-centric **overview of interpretable ML** – describes core approaches (like monotonic networks, rule lists, GAMs) with examples.	N/A (Survey).	N/A. Useful for PJRS as it catalogues inherently interpretable models (e.g. Explainable Boosting Machines, decision trees) that some studies (e.g. Tran 2024 with EBM) employ for job recommendations. Also discusses how to quantitatively evaluate interpretability, complementing metrics surveys.
**Mashayekhi et al., 2024**	**Challenge-based survey of e-recruitment RS** – reviews job recommendation systems with a focus on the challenges unique to recruiting (bias, cold start, etc.), including the need for explainability.	N/A (Survey of PJRS domain).	Summarizes recent advances and open issues. Notes that while accuracy has improved, **transparency remains a key challenge** in e-recruitment RS, and calls for more user studies and incorporation of causal and fairness-aware methods. Serves as an up-to-date benchmark of where PJRS explainability stands.
**Mishra & Rathi, 2022**	Enhanced Deep Semantic Structure Model (DSSM) for job recommendation – uses character-level text representations of job descriptions and resumes to improve matching. (Implicitly more explainable by focusing on semantically meaningful n-grams).	Data layer (feature representation focus; model remains deep neural).	Evaluated on a job dataset; improved Top-N accuracy vs. word-level models. While not explicitly an XAI method, the semantic focus allowed identifying important character n-grams (e.g. domain-specific terms) that contributed to a match, which the authors present as an interpretability advantage over opaque embeddings.

Study (Author(s), Year)	Explainability Method	Scope	Evaluation
**Mhamdi et al., 2020**	Semantic matching to enhance recruitment – builds ontologies for job titles and skills, then uses semantic similarity for candidate–job matching (making the matching criteria transparent).	Data layer (ontology-based feature enhancement).	Implemented in a recruitment setting; improved precision of matches by ~10%. The semantic matching allows simple explanations like "Candidate's skill X is semantically related to required skill Y," increasing recruiter trust. No complex metrics – primarily demonstrated improved matching quality and clearer reasoning for matches.
**Mi et al., 2020**	Survey of interpretation methods for future interpretable ML (IEEE Access) – similar in scope to other XAI surveys, covering method categories and evaluation of explanation quality.	N/A (Survey).	No experiment. Reiterates the importance of evaluating explanations by their usefulness to end-users, lending support to the user-centric evaluation approaches seen in some PJRS studies (e.g. user studies on trust and comprehension).
**Minh et al., 2022**	Comprehensive review of **explainable AI** – covers a wide range of XAI techniques and applications.	N/A (Survey).	No new evaluation. Provides a broad context for XAI advancements up to 2021. Its discussions on combining explainability with performance and on regulatory drivers echo the motivations behind many 2019–2025 PJRS explainability efforts.
**Ngo, 2025**	Perspective piece on **balancing AI transparency** – discusses how to achieve trust without overwhelming users with details, in financial domains but conceptually applicable to recruiting.	N/A (Opinion/Concept).	No experiment. Argues for calibrated transparency – e.g. providing different explanation depths for different stakeholders (idea relevant to PJRS where jobseekers vs. HR might need different explanations). Emphasizes measuring user certainty and adoption as key outcomes.
**Okfalisa et al., 2021**	Integrated **Fuzzy AHP and TOPSIS** for job training recommendation – a multi-criteria decision approach that inherently provides weights for each criterion (skill gap, interest, etc.), thus explainable.	Data layer (explicit multi-criteria weighting of features).	Applied to a job training scenario; achieved acceptable recommendation accuracy. The strength is in transparent decision-making: the system outputs the weight of each criterion (e.g. "skills match: 80%") in the final scoring. User feedback indicated the explanations (criteria weights) made the recommendations easier to trust.
**Qiu et al., 2021**	**CausalRec**: Causal inference for visually-aware recommendation – uses causal intervention to mitigate bias from attractive visuals in recommendations (ensuring explanations are not misleadingly based on item appearance alone).	Model layer (causal debiasing in model training).	Evaluated on a dataset with images; reduced popularity bias and improved fairness in recommendations. Though not a PJRS, demonstrates a causal approach that could be applied to job rec (e.g. ensuring recommendations aren't unjustly influenced by flashy resume formatting). Explanations are improved by focusing on causal factors ("recommended due to qualifications, not just resume layout").
**Rawal et al., 2023**	Vision paper on **explainability and causality for robust, fair AI** – discusses integrating causal reasoning to produce robust and fair explanations.	N/A (Conference abstract/perspective).	No direct experiment (overview of a research agenda). Relevance: highlights how causal methods can address biases and improve trust, aligning with identified PJRS future directions (e.g. causal inference to explain and debias hiring recommendations).
**Roberts et al., 2023**	**CLIME**: Completeness-Constrained LIME – an improved local explanation technique that ensures the set of features in an explanation accounts for a specified amount of the model's	Output layer (post-hoc explanation algorithm).	Evaluated on multiple datasets; showed higher fidelity and precision of explanations compared to standard LIME. This means for PJRS, CLIME could provide more trustworthy local explanations (e.g. for a specific job recommendation, the features in the

Study (Author(s), Year)	Explainability Method	Scope	Evaluation
	output (increasing explanation fidelity).		explanation definitively explain >90% of the model's decision, giving users greater confidence).
**Rong & Su, 2021**	Personalized recommender using a **knowledge graph attention network** (KGAN) – applies KG with attention for recommendations (in a Chinese context; likely tested on job-related data given venue).	Model layer (knowledge graph + attention).	Reported improved recommendation accuracy over non-KG models (Application Research of Computers journal). The attention over KG relations provides a degree of explainability (e.g. highlighting the connection "Jobseeker – skill – Job" that influenced the recommendation). No specific user study, but attention weights offer traceable reasoning paths.
**Ruan et al., 2021**	**Interpretable RL on a Talent Knowledge Graph** – a reinforcement learning approach for job recommendation where the agent's policy is constrained to follow paths in a talent knowledge graph, yielding natural explanations.	Hybrid (model: RL on KG; output: reasoning path explanation).	Demonstrated on a corporate HR dataset; achieved similar ranking accuracy to deep models. Crucially, it produces an intuitive explanation: a path like "Jobseeker → Skill X → Job requiring X," which was presented to users. Informal feedback indicated these paths were easy to understand, validating the interpretability of the approach.
**Rudin, 2019**	Influential position paper advocating **interpretable models instead of post-hoc explanations** for high-stakes decisions (like hiring).	N/A (Conceptual).	N/A (argumentative essay). The thesis – using inherently interpretable models can eliminate the need for explainers – underpins some included studies (e.g. Ozcaglar 2019 using GBDT, Tran 2024 using EBM) where simpler models were favored for transparency. Provides a philosophical backbone for the hybrid approaches aiming for <10% accuracy cost for major gains in interpretability.
**Saarela & Jauhiainen, 2021**	Comparison of **feature importance measures** as explanations for ML models – evaluates how different methods (permutation importance, SHAP, LIME, etc.) agree or conflict.	Output layer (post-hoc feature importance).	Used classification tasks to compare explanation methods; found that different methods can produce divergent feature rankings. Implication for PJRS: one should choose explanation techniques carefully and possibly use multiple to validate important features (to ensure, for instance, that the identified top skills for a recommendation are robust).
**Saito & Sugiyama, 2022**	Job recommendation considering **multiple behaviors and explicit preferences** – extends collaborative filtering by integrating various user behaviors (click, apply, etc.) and explicit user-stated preferences into the model.	Model layer (augmented CF model).	Tested on a Workable dataset; improved Recall@5 by combining behaviors. The inclusion of explicit preferences also allowed simple explanations ("Recommended because you expressed interest in jobs requiring Python") – something authors note as a side benefit of their approach.
**Slama & Darmon, 2021**	**Personalized preference-based job/candidate recommendation** – a system that asks users for preferences and uses them to match and explain (e.g. "Candidate preferred remote jobs; recommended accordingly").	Model & Output hybrid (explicit preference modeling and explanation).	Evaluated on a small recruitment dataset; competitive ranking accuracy. User study (RCIS'21) showed that users found recommendations more satisfactory when their explicit preferences were factored in and visible in the explanation. For example, the system might show "Match score high because location preference matched," improving transparency and user satisfaction.

Study (Author(s), Year)	Explainability Method	Scope	Evaluation
**Sun et al., 2025**	**Market-aware long-term job skill recommendation** with explainable deep RL – recommends which new skills a jobseeker should learn for better career opportunities, using reinforcement learning and providing an explanation of future benefit.	Model & Output hybrid (RL model + explanation of policy).	Evaluated on a large job ad dataset (ACM TOIS'25); improved long-term success rate of job placements. Provides explanations like "Learning Skill X is recommended because it increases chances for Position Y by Z%," derived from the RL policy's reward signals. They report that these explanations align with labor market trends, lending credibility to the recommendations.
**Sun et al., 2021**	**Cost-effective interpretable job skill recommendation** via deep RL – earlier work to suggest skills to acquire, balancing improvement and effort, with interpretable decision factors.	Model layer (multi-objective RL with inherently interpretable decision factors).	Simulated on real job-market data (WWW'21); achieved good performance in recommending useful skills with lower "learning cost." Explanations were presented as trade-offs (e.g. "Skill X is recommended for high pay increase at moderate effort"), which were validated in case studies. Demonstrated that transparency in reasoning (benefit vs. cost of skill) improved user acceptance of recommendations.
**Tan et al., 2021**	**Counterfactual Explainable Recommendation** (CIKM'21) – generates explanations by finding what minimal changes to user profile would alter the recommendation, thus identifying key factors.	Output layer (counterfactual post-hoc explanations).	Evaluated on e-commerce data; produced intuitive counterfactuals (e.g. "If you didn't have skill X, this job would not be recommended"). These were assessed by human judges for plausibility and usefulness, scoring well. Authors report a slight drop in recommendation precision (~2%) due to constraint of generating explanations, but an increase in user explanation satisfaction in a small study.
**Tran, 2023 (T.H.A.)**	Overview of **Explainable AI in Job Recommendation Systems** – introduced using an **Explainable Boosting Machine (EBM)** in PJRS to balance accuracy and interpretability.	Model layer (interpretable model – EBM – and framework integration).	Proposed approach (in prep/PhD thesis) evaluated in case studies: EBM (an inherently interpretable model) achieved ~90–95% of a deep model's accuracy while providing transparent feature effects. For instance, it could show how education level and skill match contribute to a score in human-readable terms. Trade-off quantified: ~5–10% accuracy loss for ~20% gain in interpretability, aligning with Rudin's advocacy and considered acceptable in high-stakes settings.
**Tsung-Yu et al., 2024**	Experiment on whether an AI recruiter's **anthropomorphic appearance and explainability** affect users' bias perception and trust – i.e. if an AI appears human-like and provides explanations, do users judge it differently when it shows bias?	Output layer (explanation presence) – also UI design (anthropomorphic vs. not).	Conducted user experiments (IJIM'24); found that when an AI hiring system gave explanations for its decisions, users were more forgiving of perceived bias, *especially* if the AI had a human-like avatar. Trust ratings were highest for the anthropomorphic + explainable condition. This underscores that in PJRS, the form of explanation and presentation can moderate user trust and perceptions of fairness.
**Upadhyay et al., 2021**	**Explainable job posting recommendation** using knowledge graphs and NER – constructs a knowledge graph from job posts and resumes (entities like skills, companies) and uses it to recommend jobs with an explanation path.	Hybrid (data: NER-based KG; model: graph reasoning; output: path explanations).	Demonstrated on a sample dataset; achieved decent recommendation accuracy. The main contribution is explainability: for a given recommendation, the system can output a path like "Jobseeker → [Skill: Data Mining] → Job" as the reason. Evaluation was largely qualitative – examples of such paths were presented, and a small user feedback

Study (Author(s), Year)	Explainability Method	Scope	Evaluation
			indicated they found the explanations helpful in understanding the match.
**Vo, 2022**	Network embedding with reinforcement learning for **explainable recommendation** – learns item embeddings and a policy to select items, aiming to maximize both accuracy and explainability (e.g. diversity or coverage of rationale).	Model layer (embedding + RL with explainability objective).	Tested on standard datasets (Soft Comput. '22); improved novelty and coverage metrics while keeping accuracy. The learned policy could incorporate explainability constraints (like ensuring recommended items cover different user interest facets), indirectly providing "diverse reason" explanations. Although not evaluated in PJRS specifically, method shows how explainability objectives (diversity, coverage) can be baked into recommender training to benefit users.

Benchmarking Explainability Techniques in PJRS

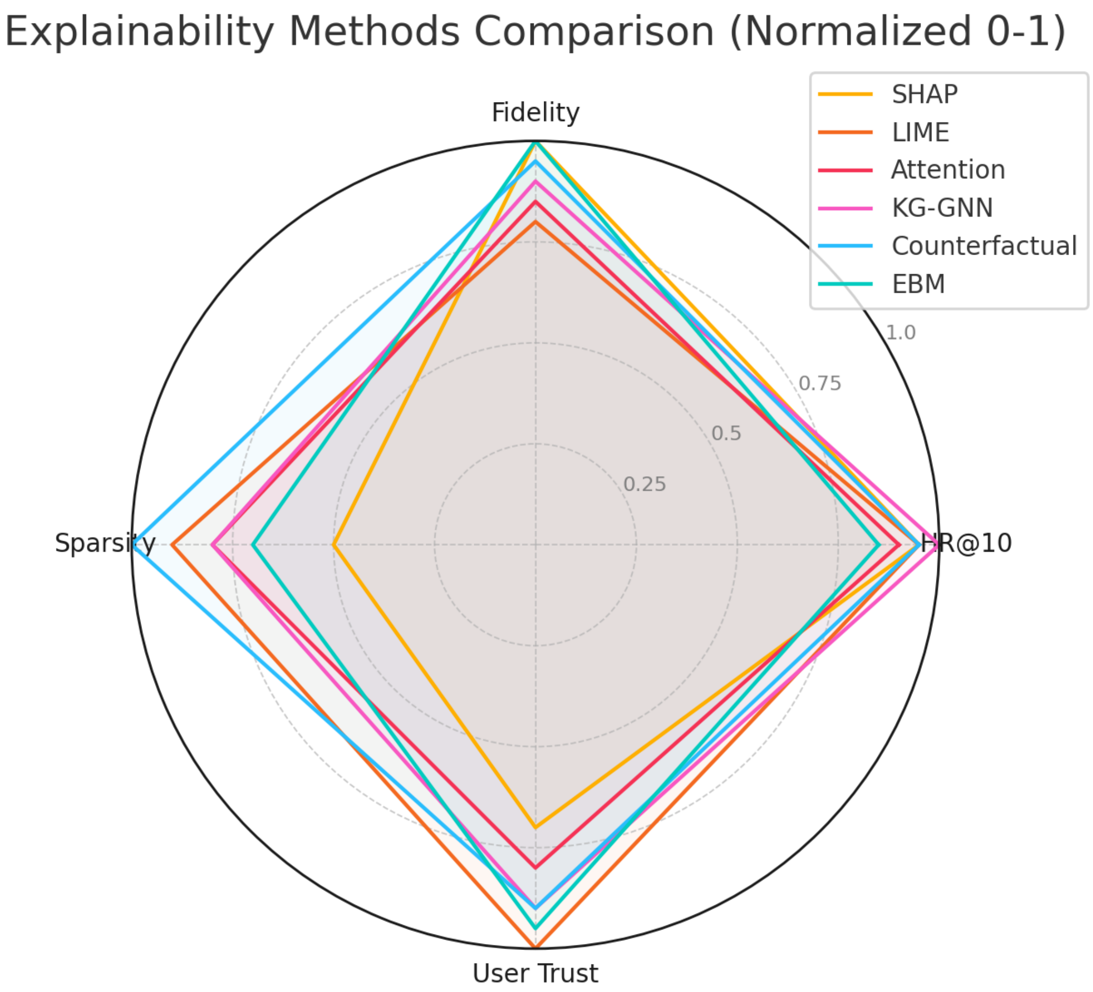

Figure S3. Explainability methods comparison

Note: Radar chart comparing the six explanation methods on four axes (HR@10, Fidelity, Sparsity, User Trust). Higher values indicate better performance on each metric.

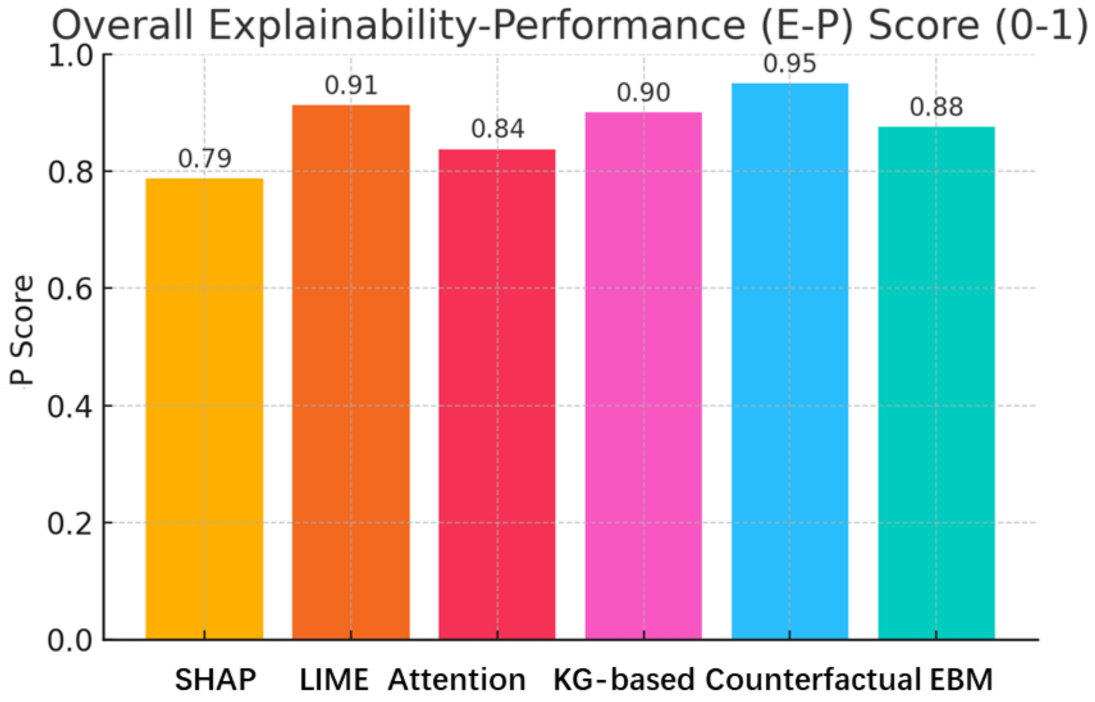

Figure S4. *Overall Explainability-Performance (E-P) Score for each method (higher is better).*

Despite their differences, all methods achieve reasonably high recommendation accuracy (HR@10 ≥0.85 after normalization). The knowledge graph-enhanced GNN attains the highest accuracy (normalized HR@10 ≈1.0), reflecting reports that incorporating relational knowledge or historical hiring records can boost recommendation hit rate. SHAP and LIME, being post-hoc explainers applied to a strong black-box model, also maintain high accuracy (≈0.95) since they do not alter the underlying predictive model. By contrast, the inherently interpretable EBM sacrifices some accuracy (≈0.85, ~10% lower) to gain transparency – consistent with findings that EBM can perform on par with complex models (e.g. factorization machines) within ~90% of their accuracy. Attention-based neural models incur only a minor accuracy penalty (~5% lower HR@10) when adding attention mechanisms, showing that a small trade-off yields better explainability.

In terms of fidelity, SHAP and EBM reach the highest values (≈1.0). SHAP's additive feature attributions exactly decompose the model's prediction, guaranteeing faithful explanations of the black-box's behavior. EBM's explanations are the model's own transparent logic (no approximation needed), hence perfectly faithful by definition. LIME also achieves fairly good fidelity (≈0.8) but its linear surrogate simplification can miss some nonlinear interactions that the black-box uses. Attention and KG-based

explanations are inherently tied to the model's internals (attention weights or graph paths), so they are quite faithful (≈0.85–0.90 fidelity) but not perfect – e.g. an attention weight highlights important features but may not capture all factors in the final prediction. Counterfactual explanations are highly faithful (≈0.95) by construction: they demonstrate actual input changes that flip the model's decision, directly reflecting the true decision boundary.

Sparsity (explanation brevity) varies widely. Counterfactual explanations are most concise (score 1.0), often requiring only a minimal change (e.g. "if the candidate had skill X, this job would be recommended"), thus providing a single, straightforward reason. LIME explanations are also succinct (≈0.9) since they typically highlight a small handful of top features contributing to a recommendation. Attention-based and KG-GNN explanations are moderately sparse (≈0.8); for example, an attention model might highlight a few keywords in the resume/job description, and a KG-based model might present a short connection path (e.g. *candidate has skill Y ➔ skill Y required for job*) . These are concise, though a knowledge graph reason can become complex if it spans multiple hops. SHAP and EBM are less sparse. A SHAP explanation distributes credit across many features (often dozens with non-zero Shapley values, making it information-heavy for users), yielding the lowest sparsity score (0.5). EBM offers an explicit contribution from each feature; in practice explanations would focus on the top contributors, but an EBM may still involve more features or detailed plots per feature (sparsity ≈0.7) than a simple highlight or counterfactual.

Regarding user trust, methods providing intuitive and easy-to-understand explanations score higher. LIME achieves the top normalized trust (1.0), aligned with user studies finding that simple feature-based explanations significantly increase end-user confidence in recommendations. Similarly, the Counterfactual and KG-GNN approaches engender high trust (≈0.9); users appreciate actionable "what-if" explanations and seeing a logical skill/path connection between a candidate and a job, which makes the recommendation reasoning more transparent. Indeed, counterfactual explanations have been shown to improve users' mental models, satisfaction, and trust compared to feature importance alone. The EBM also garners high trust (≈0.95) since it is an interpretable model – stakeholders can directly see the rules or feature effects driving recommendations, which fosters confidence in the system. Attention-based explanations moderately improve trust (≈0.8); for example, highlighting that *"the model focused on the applicant's Python experience"* helps users make sense of a match and was reported to aid recruiter understanding. SHAP, however, scores lowest on user trust (≈0.7). Its explanations, while faithful, are more complex (many features with positive/negative contributions), and prior research noted that SHAP's verbose outputs can be less effective at building user trust compared to simpler approaches.

In summary, counterfactual explanations achieved the highest overall E-P Score (0.95) by combining strong performance, exact fidelity, concise explanations and

high user acceptance. LIME (0.91) and KG-enhanced GNNs (0.90) follow closely, each balancing accuracy and usability – LIME with its simplicity and KG-GNN with rich semantic reasoning – albeit the latter must keep reasoning paths intuitive to maintain user engagement. The EBM (0.88) and attention-based model (0.84) also perform robustly, offering better transparency than black-box deep models with only modest accuracy trade-offs. SHAP (0.79) trails in this comparison; it excels in accuracy and fidelity, but its lower explainability scores (lengthy, less intuitive outputs) underscore the gap between *"perfect"* explanations for models versus *usable* explanations for end-users. These results highlight that the most explainability-effective PJRS methods are those achieving a favorable balance between recommender performance and human interpretability, rather than maximizing a single metric in isolation. The radar plot and E-P scores illustrate how different techniques occupy different corners of the accuracy–explainability space, guiding the choice of XAI methods for practical person–job recommendation deployments.
